# Lipid droplet-associated lncRNA *LIPTER* preserves cardiac lipid metabolism

**DOI:** 10.1038/s41556-023-01162-4

**Published:** 2023-06-01

**Authors:** Lei Han, Dayang Huang, Shiyong Wu, Sheng Liu, Cheng Wang, Yi Sheng, Xiongbin Lu, Hal E. Broxmeyer, Jun Wan, Lei Yang

**Affiliations:** 1 Department of Pediatrics, Herman B Wells Center for Pediatric Research, Indiana University School of Medicine, Indianapolis, IN USA; 2 Department of Medical and Molecular Genetics, Indiana University School of Medicine, Indianapolis, IN USA; 3 Department of Obstetrics, Gynecology and Reproductive Sciences, University of Pittsburgh, Pittsburgh, PA USA; 4 Department of Microbiology and Immunology, Indiana University School of Medicine, Indianapolis, IN USA

**Keywords:** Long non-coding RNAs, Cardiomyopathies, Organelles

## Abstract

Lipid droplets (LDs) are cellular organelles critical for lipid homeostasis, with intramyocyte LD accumulation implicated in metabolic disorder-associated heart diseases. Here we identify a human long non-coding RNA, Lipid-Droplet Transporter (*LIPTER*), essential for LD transport in human cardiomyocytes. *LIPTER* binds phosphatidic acid and phosphatidylinositol 4-phosphate on LD surface membranes and the MYH10 protein, connecting LDs to the MYH10-ACTIN cytoskeleton and facilitating LD transport. *LIPTER* and *MYH10* deficiencies impair LD trafficking, mitochondrial function and survival of human induced pluripotent stem cell-derived cardiomyocytes. Conditional *Myh10* deletion in mouse cardiomyocytes leads to LD accumulation, reduced fatty acid oxidation and compromised cardiac function. We identify *NKX2.5* as the primary regulator of cardiomyocyte-specific *LIPTER* transcription. Notably, *LIPTER* transgenic expression mitigates cardiac lipotoxicity, preserves cardiac function and alleviates cardiomyopathies in high-fat-diet-fed and *Lepr*^db/db^ mice. Our findings unveil a molecular connector role of *LIPTER* in intramyocyte LD transport, crucial for lipid metabolism of the human heart, and hold significant clinical implications for treating metabolic syndrome-associated heart diseases.

## Abstract (web summary)

Han et al. identify the long non-coding RNA *LIPTER* as a key mediator of lipid droplet transport and metabolism in human cardiomyocytes. *LIPTER* overexpression mitigates cardiomyopathy and preserves cardiac function in obese and diabetic mouse models.

## Main

Lipid droplets (LDs) are highly dynamic cellular organelles ubiquitously existing in prokaryotes and eukaryotic cells. Synthesized in the endoplasmic reticulum (ER), LDs consist of a neutral lipid core, primarily containing triacylglycerols (TAGs) and sterol esters, surrounded by a phospholipid monolayer with associated proteins^1^. Although initially considered storage reservoir for excess lipids, LDs have been increasingly recognized for their critical roles in intracellular lipid trafficking, lipid homeostasis and membrane synthesis^1,2^. Under hyperlipidaemic conditions, LDs accumulate in the myocardium (that is, cardiac steatosis) and cardiomyocytes (CMs)^2^. While increased LD levels afford transient cardiac protection by storing surplus fatty acids (FAs) in CMs^3,4^, long-term lipid intermediary accumulation results in detrimental outcomes, such as apoptosis, tissue injury and cardiac dysfunction (that is, lipotoxicity)^2,5,6^. Intramyocyte LD accumulation in humans is associated with cardiomyopathies and heart failure in hyperlipidaemia-related metabolic disorders, including obesity and diabetes mellitus^6,7^. Animal model studies have also revealed that an expanded pool of LDs can lead to early impairment of cardiac contractility and subsequent heart dysfunction^8–10^. Despite these findings, the CM intrinsic mechanisms determining LD accumulation and mobilization, as well as the aetiology of intramyocyte LD accumulation in obesity and diabetes mellitus, remain poorly understood.

LDs play a vital role in cellular metabolism, particularly under nutrient-deprived conditions. Under starvation conditions, LDs are mobilized and transported to mitochondria for β-oxidation to generate energy for the cell^11^. TAG within LD core is digested to FAs via lipolysis and lipophagy processes on LDs^1,12^. The extensive interactions of LDs with various organelles, such as the ER, endosomes and mitochondria, facilitate intracellular lipid trafficking and channelling^13^. LD-associated proteins mediate LD–organelle membrane contacts. For example, FATP1 and DGAT2 at the LD–ER interface regulate LD expansion^14^, while LD–mitochondria tethering in skeletal and heart muscle is mediated by PLIN5 for channelling FAs from LDs to mitochondria^15^. Although LD biogenesis, composition and turnover have been extensively studied, the molecular mechanism underlying LD transport from the ER to other organelles remains elusive. Previous studies have implicated myosin motors, such as Myosin I and V, in the transport of ER-derived vesicles^16,17^. Additionally, *Myh9* depletion has been shown to impair LD dissociation from the ER^18^. Mammalian CMs predominantly express non-muscle myosin IIB (Myh10) (ref. ^19^), which can interact with membrane lipids^20^ and link cytoskeleton with the ER^21^. While the key roles of protein factors in intracellular LD trafficking have been extensively studied, the direct involvement of RNA molecules in this process remains unknown. Gaining insight into this aspect is crucial for understanding intramyocyte lipid trafficking and channelling, which are essential for maintaining lipid homeostasis in the human heart.

Although RNA is primarily known for carrying and regulating genetic information, recent evidence indicates that RNA interacts with various molecules, such as nucleic acids, ions, proteins and lipids^22^, to perform diverse functions. RNA–lipid interactions have revealed new regulatory mechanisms in essential cellular processes, implying previously undefined functions of RNA. In this study, we identified a human long non-coding RNA (lncRNA), named Lipid-Droplet Transporter (*LIPTER)*, which facilitates LD transport within human CMs. *LIPTER* deficiency impairs LD transport and metabolism, mitochondrial function and CM viability. Mechanistically, *LIPTER* directly binds two phospholipids on LD membrane, phosphatidic acid (PA) and phosphatidylinositol 4-phosphate (PI4P), as well as the MYH10 motor protein, connecting LDs with cytoskeleton for intramyocyte transport. Importantly, *LIPTER* overexpression mitigates cardiomyopathies and preserves cardiac functions in mouse models of obesity and diabetes, highlighting *LIPTER*’s potential clinical relevance in treating human metabolic syndrome-associated heart disease and failure. Our findings reveal that RNA can directly participate in intracellular vesicle transport, expanding RNA’s functional dimensions and suggesting a potential ancient role of RNA–lipid interaction in forming the first living system in the primordial ‘RNA world’.

## Results

### *LINC00881* (*LIPTER*) is specifically expressed in human CMs

**Fig. 1:**
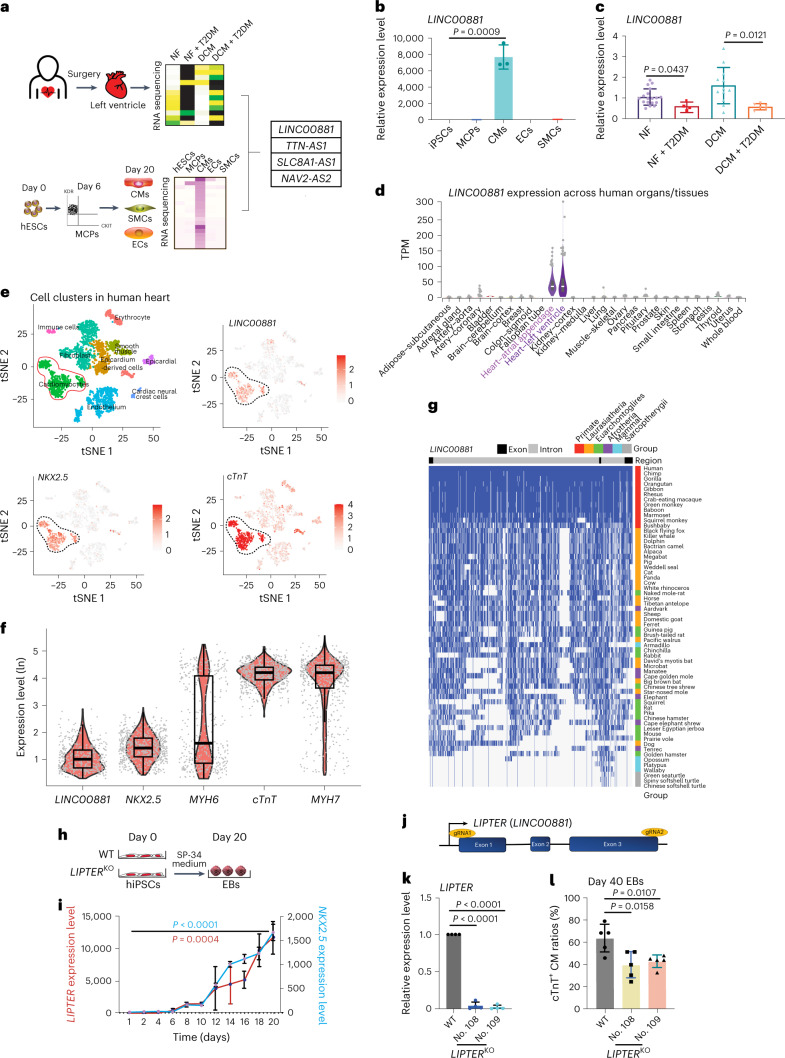
Identification of a human CM-specific lncRNA *LIPTER* (*LINC00881*). **a**, A schematic diagram shows the integrated transcriptomic analyses of data from human heart tissues and human embryonic stem cell (hESC)-derived cardiovascular cells. NF, non-failure. **b**, RT–qPCR detection of *LINC00881* expression in hiPSC-derived cardiovascular cell types (*n* = 3 independent experiments). **c**, RT–qPCR results of *LINC00881* expressions in all collected human hearts. NF (*n* = 18 samples), NF + T2DM (*n* = 4 samples), DCM (*n* = 12 samples), DCM + T2DM (*n* = 6 samples). **d,** *LINC00881* expression profile across human organs from the NIH Genotype-Tissue Expression (GTEx) project database. TPM, transcripts per million. Whiskers show the minimum to maximum values, and bounds of boxes represent first and third quantiles and the centre line indicates the median. **e**, The scRNA-seq data from a human foetal heart reveal CM-specific *LINC00881* expression. **f**, Violin plots showing *LINC00881* (*n* = 439 cells), *NKX2.5* (*n* = 647 cells), *MYH6* (*n* = 417 cells), *CTNT* (*n* = 718 cells) and *MYH7* (*n* = 582 cells) expression in CMs by analysing scRNA-seq data from **e**. Whiskers show the minimum to maximum values, and bounds of boxes represent first and third quantiles and the centre line indicates the median. **g**, Interspecies conservation analysis of *LINC00881* sequence. **h**, A scheme of CM differentiation from hiPSCs by forming EBs. **i**, Expression dynamics of *LIPTER* and *NKX2.5* during CM differentiation. Dots are presented as mean ± s.e.m. Unpaired two-tailed *t*-test is used for comparison (*n* = 3 independent experiments). **j**, Dual gRNAs were designed to completely knock out *LIPTER* in hiPSCs using CRISPR/Cas-9. **k**, *LIPTER* expression in WT and *LIPTER*^KO^ hiPSC-derived EBs at day 20 (*n* = 4 independent experiments). **l**, Ratios of cTnT^+^ CMs in day 40 hiPSC-EBs (*n* = 4 independent experiments). In **b**, **c**, **k** and **l**, bars are represented as mean ± s.e.m. Unpaired two-tailed *t*-test is used for comparison. Source numerical data are available in source data. Source data

**Extended Data Fig. 1:**
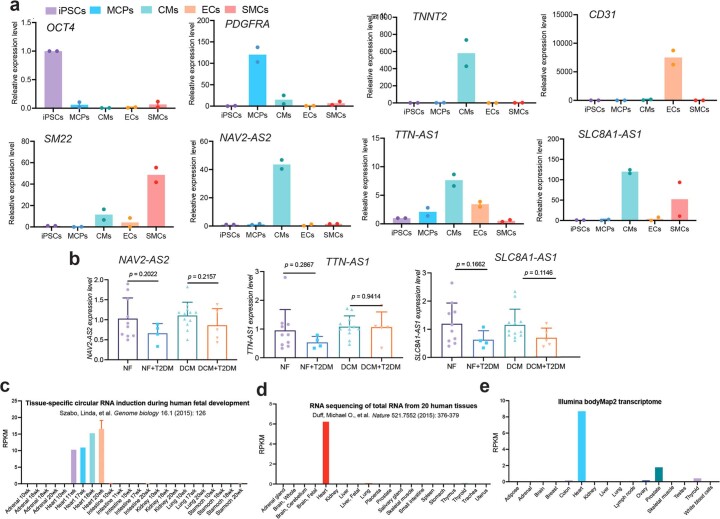
Identification of human CM-specific lncRNA *LIPTER* (*LINC00881*). **a**, RT-qPCR detection of pluripotency marker *OCT4*, cardiac progenitor marker *PDGFRA*, cardiomyocyte marker *TNNT2*, endothelial cell marker *CD31*, smooth muscle cell marker *SM22*, as well as lncRNAs *NAV2-AS2*, *TTN-AS1* and *SLC8A1-AS1* expressions in enriched cardiovascular cell types differentiated from human iPS cells. (n = 2 independent experiments). **b**, RT-qPCR detection of lncRNAs *NAV2-AS2*, *TTN-AS1* and *SLC8A1-AS1* expression levels in left ventricle tissues from NF (n = 10 samples), NF+T2DM (n = 4 samples), DCM (n = 12 samples) and DCM+T2DM (n = 5 samples) individuals. All bars are presented as mean ± s.e.m. Unpaired two-tailed *t-test* is used for comparison. **c**, Histogram of *LINC00881* expressions in the human hearts during fetal development. RPKM, Reads Per Kilobase of transcript (RPKM). Bars are presented as mean ± sd. Data were analyzed from GSE64283^73^. **d**, Histogram of *LINC00881* expressions across 20 human tissues. Data were analyzed from NCBI Accession PRJNA280600^74^. **e**, Histogram of *LINC00881* expressions across 16 human tissues. Data were analyzed from GSE30611 (Illumina Human Body Map 2.0 Project). Source numerical data are available in source data. Source data

**Extended Data Fig. 2:**
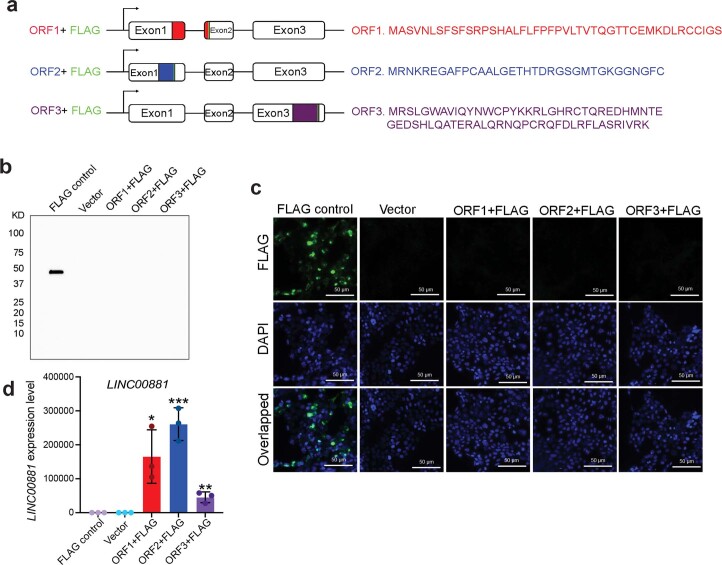
*LINC00881* contains no coding potential. **a**, ORFs predicted in *LINC00881*, showing predicted peptide sequence of each ORF. A FLAG tag was inserted right after each ORF. **b**, Western blotting detection of peptide expressions in HEK293T cells transfected with positive control, vector, and FLAG tagged ORF1, ORF2 or ORF3. Positive control is an irrelevant FLAG tagged protein coding gene. The experiment was carried out 2 times with similar outcomes. **c**, Immunofluorescent staining to detect expressions of FLAG tagged peptides in HEK293T cells transfected with positive control, vector, and FLAG tagged ORF1, ORF2 or ORF3. **d**, RT-qPCR detecting *LINC00881* RNA expression levels in HEK293T cells transfected with positive control, vector, and FLAG tagged ORF1, ORF2 or ORF3. Bars are presented as mean ± s.e.m. Unpaired two-tailed *t-test* is used for comparison. **p < 0.01, ***p < 0.001, (n = 3 independent experiments). Source numerical data and unprocessed blots are available in source data. Source data

Using our previously established method^23^, human pluripotent stem cells (hPSCs) were differentiated into cardiovascular cells. Transcriptomic profiles of hPSCs, hPSC-derived multipotential cardiovascular progenitors (MCPs), CMs, smooth muscle cells (SMCs), endothelial cells (ECs)^24^ and left ventricle tissues from individuals with and without type 2 diabetes (T2DM) (3 individuals per group) were compared. We identified the top four CM-enriched lncRNAs, *LINC00881*, *TTN-AS1*, *SLC8A1-AS1* and *NAV2-AS2*, which were downregulated in T2DM hearts compared with non-T2DM hearts (Fig. 1a,b and Extended Data Fig. 1a). However, RT–qPCR validation confirmed that only *LINC00881* expression was significantly reduced to less than 50% (*P* < 0.05) in all collected T2DM hearts compared with non-T2DM hearts (Fig. 1c and Extended Data Fig. 1b). Multiple tissue-specific gene expression databases demonstrated that *LINC00881* expression is highly enriched in the human hearts (Fig. 1d and Extended Data Fig. 1c–e). Additionally, the analysis of single-cell RNA sequencing (scRNA-seq) data from a 6.5–7 week human embryonic heart^25^ revealed that *LINC00881* expression was specifically enriched in the *NKX2.5*^*+*^ and cardiac Troponin T (*cTnT*)^*+*^ CM clusters (Fig. 1e), with an expression level comparable to the key cardiac transcription factor (TF) *NKX2.5* (ref. ^26^) (Fig. 1f). *LINC00881* is conserved in human and non-human primates but not across other species (Fig. 1g). Although three putative open reading frames (ORFs) were predicted in *LINC00881* by ORF-FINDER^27^ (Extended Data Fig. 2a), the precited peptides were not detected using western blotting or immunostaining (Extended Data Fig. 2b,c), albeit increased RNA level of *LINC00881* detected in transfected 293T cells (Extended Data Fig. 2d). Altogether, these results demonstrate that *LINC00881* is highly and specifically enriched in human CMs and significantly downregulated in human T2DM hearts. In this study, *LINC00881* was renamed *LIPTER* due to its role in LD transport within human CMs.

### *LIPTER* deficiency impairs LD metabolism and transport

**Fig. 2:**
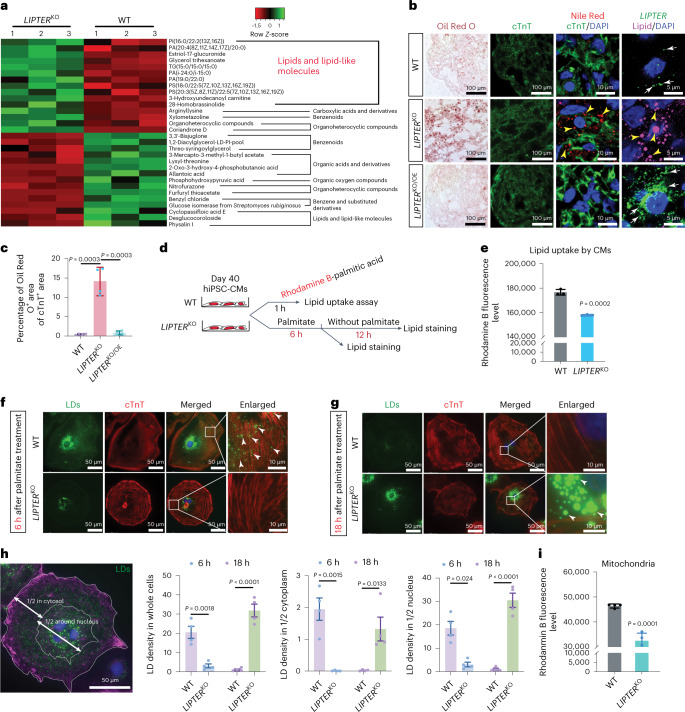
*LIPTER* deficiency disrupts LD balance of hiPSC-CMs. **a**, Heat map showing top changed metabolites in *LIPTER*^KO^ versus WT hiPSC-CMs at day 40 of differentiation. **b**, Oil Red O lipid staining and cTnT immunostaining (first two columns); Nile Red and cTnT co-staining (third column); *LIPTER* RNA FISH and Lipid Deep Red co-staining (fourth column) in WT, *LIPTER*^KO^ and *LIPTER*^KO/OE^ hiPSC-CMs. **c**, Quantification of the ratios of Oil Red O^+^ areas to cTnT^+^ CM areas (*n* = 4 independent experiments). **d**, Schematic of lipid uptake and LD mobilization analysis in palmitic acid (palmitate)-treated WT and *LIPTER*^KO^ hiPSC-CMs. **e**, Uptake of Rhodamine B-palmitic acid by whole hiPSC-CMs (*n* = 3 independent experiments). **f**,**g**, Representative fluorescence images of LD accumulation/distribution in WT and *LIPTER*^KO^ hiPSC-CMs treated with 200 μM palmitate for 6 h (**f**), followed by palmitate depletion for an additional 12 h (**g**). **h**, Quantification of relative LD densities in whole CMs, and in 1/2 cytosolic and 1/2 nucleus surrounding areas in CMs (*n* = 4 independent experiments). **i**, Measurement of Rhodamine B fluorescence levels in mitochondria isolated from hiPSC-CMs post treatment with Rhodamine B-palmitic acid for 2 h (*n* = 4 independent experiments). In **c**, **e**, **h** and **i**, bars are presented as mean ± s.e.m. Unpaired two-tailed *t*-test is used for comparison. Source numerical data are available in source data. Source data

**Extended Data Fig. 3:**
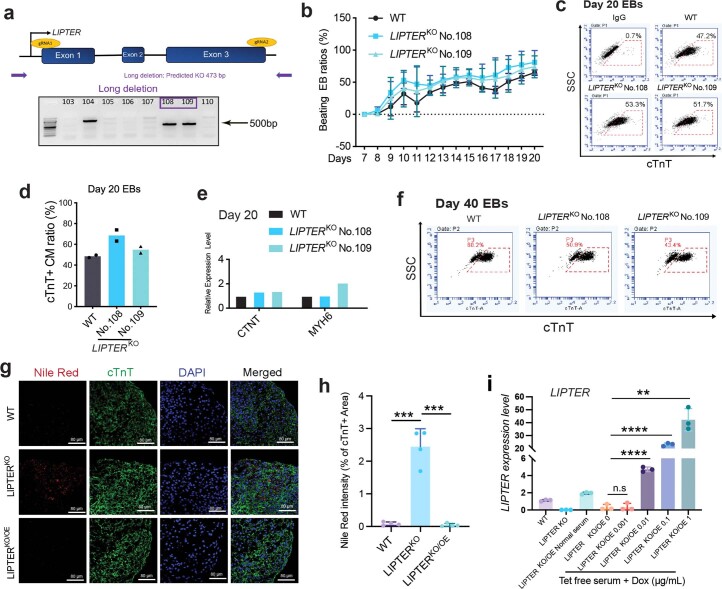
*LIPTER* deficiency impairs survival of long-term cultured hiPSC-CMs. **a**, *LIPTER* was knocked out in hiPSCs using CRISPR /Cas-9. Dual gRNAs were designed to completely ablate *LIPTER*. Genotyping was carried out in individual hiPSC clones using PCR primer sets for detecting long deletions. **b**, Ratios of beating EBs during CM differentiation from WT and two *LIPTER*^KO^ hiPSC lines. Dots represent mean values ± s.e.m. Unpaired two-tailed *t-test* is used for comparison. No significance detected. (n = 3 independent experiments). **c**, Representative FACS results of cTnT^+^ cells ratios at day 20 differentiation of WT and *LIPTER*^KO^ hiPSC lines. **d**, Quantification of cTnT^+^ cell ratios at day 20 of differentiation. Bars represent mean values, (n = 2 independent experiments). **e**, RT-qPCR detecting expressions of CMs markers *CTNT*, *MYH6* and *MYH7* in WT and *LIPTER*^KO^ hiPSC-EBs at day 20 of differentiation. Bars represent mean values, (n = 2 independent experiments). **f**, Representative FACS results of cTnT^+^ CM ratios in WT and *LIPTER*^KO^ hiPSC-EBs at day 40 of differentiation. **g**, Nile Red and cTnT co-staining in WT, *LIPTER*^KO^ and *LIPTER*^KO/OE^ hiPSC-derived EB sections. **h**, Quantification of Nile Red positive areas in cTnT^+^ CM areas. Bars represent mean values ± s.e.m. Unpaired two-tailed *t-test* is used for comparison. ***p < 0.001. (n = 4 independent experiments). **i**, RT-qPCR detection of *LIPTER* overexpression levels in *LIPTER*^KO/OE^ hiPSC-CMs treated with various concentrations of doxycycline for 4 days. Bars represent mean values ± s.e.m. Unpaired two-tailed *t-test* is used for comparison. ****p < 0.0001, **p < 0.01, n.s (no significance). (n = 3 independent experiments). Source numerical data are available in source data. Source data

During CM differentiation from human induced pluripotent stem cells (hiPSCs) by forming embryoid bodies^23^ (EBs; Fig. 1h), *LIPTER* expression rapidly increased by over 10,000-fold (CMs versus hiPSCs), similar to *NKX2.5* (Fig. 1i). Next, two *LIPTER* knockout (*LIPTER*^KO^) hiPSC clones were established by CRISPR/Cas-9 (ref. ^28^) (Fig. 1j,k and Extended Data Fig. 3a). *LIPTER*^KO^ did not affect the ratios of beating EBs and cTnT^+^ CMs or the expression levels of CM markers, *cTnT* and *MYH6*, at day 20 of CM differentiation (Extended Data Fig. 3b–e), indicating that *LIPTER* is not required for CM differentiation. However, since *LIPTER* expression declines in T2DM hearts (Fig. 1c), we cultured hiPSC-EBs with high glucose (22.75 mM) for an additional 20 days. At day 40, significantly reduced ratios of cTnT^+^ CMs were observed in *LIPTER*^KO^ compared with wild-type (WT) hiPSC-EBs (Fig. 1l and Extended Data Fig. 3f), suggesting that *LIPTER* is required for CM survival under high-glucose condition and implicating a potential role of *LIPTER* in CM metabolism. To investigate global changes in metabolites upon *LIPTER* deficiency, untargeted metabolomics (Fig. 2a and Supplementary Table 1) were performed on enriched day 40 hiPSC-CMs. The top increased metabolites in *LIPTER*^KO^ compared with WT hiPSC-CMs were lipid and lipid-like molecules, including phosphatidylinositol (PI), PAs and TAGs. Notably, TAG is the main core lipid of LDs^1,29^, while PI and PA are phospholipids on LD membranes^29^. These data suggest increased LD levels in *LIPTER*^KO^ compared with WT hiPSC-CMs, which was confirmed by Oil Red O and Nile Red lipid staining (*LIPTER*^KO^ versus WT; Fig. 2b,c and Extended Data Fig. 3g,h). Finally, *LIPTER* expression was rescued in *LIPTER*^KO^ (*LIPTER*^KO/OE^) hiPSCs using doxycycline-inducible lentivirus. After CM differentiation, *LIPTER* expression was induced, resulting in a two- to fourfold increase compared with WT hiPSC-CMs (Extended Data Fig. 3i). We observed that rescued *LIPTER* significantly repressed LD accumulation in *LIPTER*^KO^ hiPSC-CMs (*LIPTER*^KO/OE^ versus *LIPTER*^KO^; Fig. 2b,c and Extended Data Fig. 3g,h). Collectively, these data demonstrate that *LIPTER* deficiency disrupts lipid metabolism and enhances LD accumulation in hiPSC-CMs.

**Extended Data Fig. 4:**
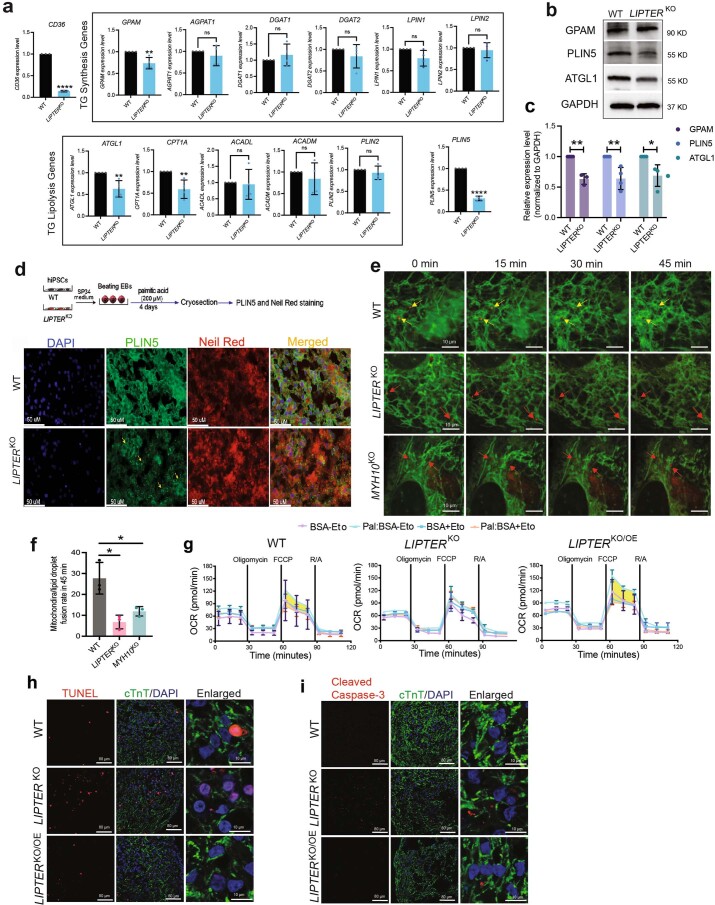
*LIPTER* deficiency compromises mitochondrial function and induces apoptosis of human iPSC-CMs. **a**, Comparing the expression levels of *CD36*, TG synthesis/lipolysis related genes and *PLIN5* in enriched WT and *LIPTER*^KO^ hiPSC-CMs at day 40 of differentiation using RT-qPCR. Bars represent mean values ± s.e.m. Unpaired two-tailed *t-test* is used for comparison. ****p < 0.0001, **p < 0.01. ns. no significance. (n = 4 independent experiments). **b**, Western blotting detection of GPAM, PLIN5 and ATGL1 protein expressions in enriched WT and *LIPTER*^KO^ hiPSC-CMs at day 40 of differentiation. **c**, Statistical analysis of Western blotting results in **b**. Bars represent mean values ± s.e.m. Unpaired two-tailed *t-test* is used for comparison. **p < 0.01, *p < 0.05. (n = 3 times of independent experiments in the 1^st^ and 3^rd^ groups; n = 4 independent experiments in the 2^nd^ group). **d**, WT and *LIPTER*^KO^ hiPSC-derived embryoid bodies were treated with 200 µM palmitic acid for 4 days, followed by immunostaining for PLIN5 and Neil Red lipid staining on EB sections. Arrows indicate condensed PLIN5. The experiment was carried out 3 times with similar outcomes. **e**, Live cell images showing the fusion of Rhodamine B-palmitic acid (red) labeled-LDs with mitochondria (green) and subsequent diminishment in WT hiPSC-CMs over 45 min, while no apparent LD-mitochondria fusion was observed in *LIPTER*^KO^, and *MYH10*^KO^ hiPSC-CMs. Images were from the continuous live cell imaging during 45 min of cultured hiPSC-CMs. The experiment was carried out 3 times with similar outcomes with statistical results in (**f**). **f**, Quantification of the ratios of LD fused with mitochondria in **e**. Bars represent mean values ± s.e.m. Unpaired two-tailed *t-test* is used for comparison. *p < 0.05. (n = 3 independent experiments). **g**, Analyses of the maximal oxygen consumption values with Palmitate:BSA between conditions without and with Etomoxir (Eto) to compare relative fatty acid oxidation (FAO) capabilities (yellow zone) in WT, *LIPTER* ^KO^ and *LIPTER* ^KO/OE^ hiPSC-CMs. The experiment was carried out 4 times with similar outcomes. **h**, Representative immunofluorescent images of TUNEL and cTnT co-staining on day 40 hiPSC-EB sections. The experiment was carried out 5 times with similar outcomes. **i**, Representative immunofluorescent images of Cleaved Caspase-3 and cTnT co-staining on day 40 hiPSC-EB sections. The experiment was carried out 4 times with similar outcomes. Source numerical data and unprocessed blots are available in source data. Source data

Intramyocyte LD accumulation could be due to increased uptake of free FAs via CD36, a membrane receptor to import extracellular FAs^30^. However, both CD36 expression (Extended Data Fig. 4a) and lipid uptake capability were reduced in *LIPTER*^KO^ hiPSC-CMs compared with WT hiPSC-CMs (Fig. 2d,e), indicating that LD accumulation was not a result of enhanced FA uptake. We detected no significant changes in genes for TAG synthesis and lipolysis on LDs, except for ~30% reductions of *GPAM* and *ATGL1* (refs. ^1,31^) (Extended Data Fig. 4a–c), upon *LIPTER*^KO^, suggesting that accumulated LDs in *LIPTER*^KO^ CMs might not be primarily caused by altered LD formation or lipolysis process. Interestingly, *LIPTER*^KO^ prominently reduced expression and induced accumulation of *PLN5* (Extended Data Fig. 4a–d), which was reported to mediate LD–mitochondria tethering in CMs^15^. These results implied that *LIPTER*^KO^ could impair LD transport to and/or interaction with mitochondria.

To trace LD transport, hiPSC-CMs were cultured with palmitate (200 μM) for 6 h to induce LD formation (Fig. 2d). In WT hiPSC-CMs, LDs surrounded nucleus and were broadly distributed in the cytoplasm (Fig. 2f, top, arrowheads), while cytosolic transport and distribution of LDs were retarded in *LIPTER*^KO^ hiPSC-CMs (Fig. 2f,bottom). We then cultured CMs without palmitate for additional 12 h to mobilize the LDs (Fig. 2d). While LDs in WT hiPSC-CMs were fully mobilized, large LDs accumulated in *LIPTER*^KO^ hiPSC-CMs (Fig. 2g, arrowheads). Statistical results show that, from 6 h to 18 h, LD densities in WT hiPSC-CMs rapidly declined in whole CMs, cytoplasm and the surrounding region of nucleus, whereas high amounts of LDs remained in *LIPTER*^KO^ hiPSC-CMs (Fig. 2h). Since mobilized LDs could transfer stored lipids to mitochondria for β-oxidation^1,2^, we conducted live cell imaging to trace LD–mitochondria interaction. Rhodamine-B-labelled palmitic acid was added to CM culture for 6 h and enriched in LDs. In WT hiPSC-CMs, approximately 28% of LDs^Rh-B^ fused with mitochondria and then disappeared (Extended Data Fig. 4e, top, yellow arrows; Extended Data Fig. 4f), whereas only about 8% of LDs^Rh-B^ fused with mitochondria in *LIPTER*^KO^ hiPSC-CMs (Extended Data Fig. 4e, middle, red arrows; Extended Data Fig. 4f). Finally, mitochondria were isolated from hiPSC-CMs after 2 h of Rhodamine-B-labelled palmitic acid treatment. Significantly reduced fluorescence levels were detected in *LIPTER*^KO^ versus WT mitochondria (Fig. 2i), indicating reduced Rhodamine-B-labelled palmitic acid transfer to mitochondria upon *LIPTER* ablation. Collectively, these results demonstrate that *LIPTER* deficiency disrupts LD transport and mobilization in human CMs, leading to extensive LD accumulation.

### *LIPTER* deficiency induces mitochondrial dysfunction and apoptosis

**Fig. 3:**
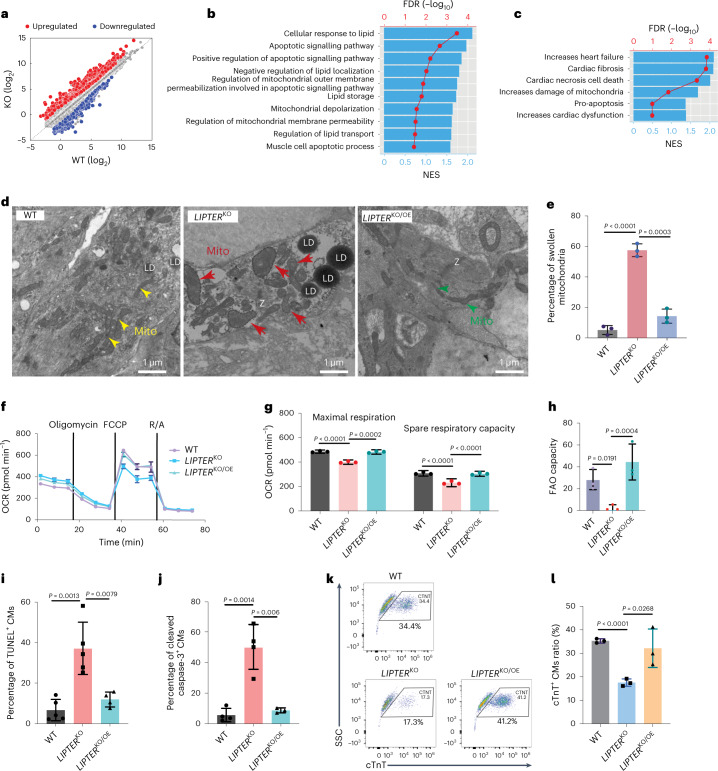
*LIPTER* deficiency results in global injuries of hiPSC-CMs. **a**, Whole mRNA-seq analysis showing differential gene expression profiles in *LIPTER*^KO^ versus WT hiPSC-CMs (for details, see Supplementary Table 2). **b**, Significantly changed genes upon *LIPTER*^KO^ are enriched in GO biological processes identified by GSEA analysis. NES, normalized enrichment scores. **c**, Significantly changed genes in *LIPTER*^KO^ versus WT hiPSC-CMs are enriched in cell toxicity categories. **d**, TEM images of mitochondrial morphologies in WT, *LIPTER*^KO^ and *LIPTER*^KO/OE^ hiPSC-CMs. Yellow and green arrows indicate normal mitochondria; red arrows indicate swollen mitochondria; Z, Z-band. *LIPTER*^KO/OE^, rescued *LIPTER* overexpression in *LIPTER*^KO^. The experiment was carried out three times with similar outcomes. **e**, Quantification of swollen mitochondria ratios from **d** (*n* = 3 independent experiments). **f**, Mitochondrial OCR measurement in hiPSC-CMs. **g**, Analyses of maximum respiratory capacity and spare respiratory capacity in OCR, normalized to total protein content of each well (*n* = 3 independent experiments). **h**, Quantification of FAO rates. **i**, Ratios of TUNEL^+^ CMs in day 40 hiPSC-EBs (*n* = 5 in the first 2 groups and *n* = 4 in the last group of independent experiments). **j**, Ratios of cleaved caspase-3^+^ CMs in day 40 hiPSC-EBs (*n* = 4 in the first 2 groups and *n* = 3 in the last group of independent experiments). **k**, FACS analysis of cTnT^+^ CM ratios in day 50 hiPSC-EBs, with statistical results in **l** (*n* = 3 independent experiments). In **e**, **g**–**j** and **l**, bars are presented as mean ± s.e.m. Unpaired two-tailed *t*-test is used for comparison. Source numerical data are available in source data. Source data

Next, we performed whole messenger RNA sequencing (mRNA-seq) (Supplementary Table 2) and discovered that *LIPTER*^KO^ altered the transcriptome of hiPSC-CMs (Fig. 3a). The genes with significant expression changes were enriched into Gene Ontology (GO) pathways, including elevated apoptotic signalling, aberrant mitochondrial functions and lipid storage (Fig. 3b), as well as toxicity signalling pathways, such as increased heart failure and cardiac dysfunction (Fig. 3c). Consistently, transmission electron microscopy (TEM) revealed compact and rod-shaped mitochondria in WT hiPSC-CMs (Fig. 3d, yellow arrowheads), while ~60% of mitochondria in *LIPTER*^KO^ hiPSC-CMs displayed giant and/or swollen morphology (Fig. 3d, red arrows, Fig. 3e), indicating mitochondrial dysfunction. Mass spectrometry (MS) of purified mitochondria revealed that *LIPTER*^KO^ altered the expressions of mitochondrial respiratory proteins, such as reduced levels of NDUFB2, NDUFB11 and COX7B (Supplementary Table 3). Compared with WT hiPSC-CMs, mitochondrial maximal respiration capacity, spare respiratory capacity (Fig. 3f,g) and long-chain fatty acid oxidation (FAO) capacities of *LIPTER*^KO^ hiPSC-CMs (Fig. 3h and Extended Data Fig. 4g) were significantly reduced. Furthermore, increased apoptosis was detected in *LIPTER*^KO^ versus WT hiPSC-CMs (Fig. 3i,j and Extended Data Fig. 4h,i). To investigate whether these CM abnormalities were *LIPTER* dependent, *LIPTER* expression was rescued in *LIPTER*^KO^ hiPSC-CMs, which restored the swollen and dysfunctional mitochondria and suppressed apoptosis of *LIPTER*^KO^ CMs (Fig. 3i,j, *LIPTER*^KO/OE^ versus *LIPTER*^KO^). Consequently, rescued *LIPTER* enhanced the ratios of cTnT^+^ CMs at day 50 of EB differentiation compared with *LIPTER*^KO^ (Fig. 3k,l). Altogether, these results reveal the crucial roles of *LIPTER* in mitochondrial function and viability of human CMs.

### *NKX2.5* controls CM-specific *LIPTER* transcription

**Fig. 4:**
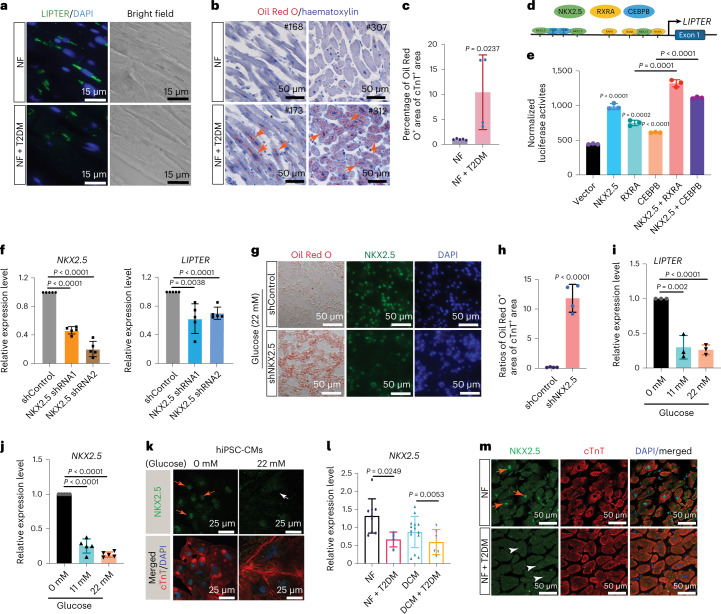
*NKX2.5* controls CM-specific *LIPTER* transcription and its downregulation in diabetic hearts. **a**, RNA FISH detecting *LIPTER* in human heart tissues. NF, non-failure; NF + T2DM, non-failure with T2DM. **b**, Oil Red O staining to detect LDs in human heart tissues. **c**, Quantification of Oil Red O^+^ areas in cTnT^+^ areas. NF (*n* = 5 samples), NF + T2DM (*n* = 4 samples). **d**, PROMO, UCSC Genome Browser and TFBIND algorithms predict TF binding sites on the *LIPTER* promotor region. **e**, Dual luciferase reporter assay measuring relative *LIPTER* promoter activities driven by TFs, normalized to Renilla luciferase activity (*n* = 3 independent experiments). **f**, RT–qPCR detection of *LIPTER* and *NKX2.5* expressions in WT hiPSC-CMs infected with AAV9-shControl or AAV9-shRNAs against *NKX2.5* for 2 weeks (*n* = 5 independent experiments). **g**, Representative images of Oil Red O and NKX2.5 staining of hiPSC-CMs infected with AAV9-shControl or AAV9-shRNA against *NKX2.5* under high-glucose (22 mM) conditions. **h**, Quantification of Oil Red O^+^ areas in cTnT^+^ CM areas (*n* = 4 independent experiments). **i**,**j**, RT–qPCR results of *LIPTER* (**i**, *n* = 3 independent experiments) and *NKX2.5* (**j**, *n* = 5 independent experiments) expression in WT hiPSC-CMs treated with high-glucose conditions for 2 weeks. **k**, Representative immunofluorescent images of NKX2.5 staining in WT hiPSC-CMs treated with 0 and 22 mM glucose for 2 weeks. **l**, RT–qPCR results of *NKX2.5* mRNA levels in human left ventricle tissues. NF (*n* = 9 samples), NF + T2DM (*n* = 4 samples), DCM (*n* = 14 samples), DCM + T2DM (*n* = 6 samples). **m**, Representative fluorescence images of NKX2.5 staining in human left ventricle tissues from three samples per condition. In **c**, **e**, **f**, **h**–**j** and **l**, bars are presented as mean ± s.e.m. Unpaired two-tailed *t*-test is used for comparison. Source numerical data are available in source data. Source data

**Extended Data Fig. 5:**
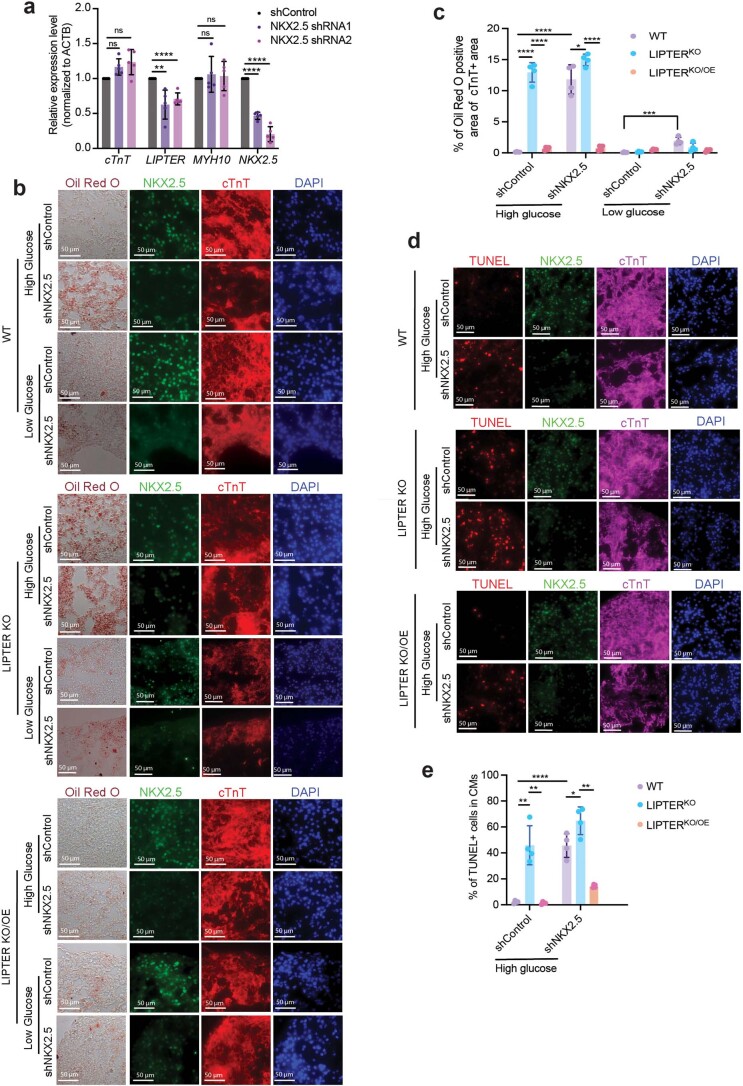
*NKX2.5* deficiency reduces *LIPTER* expression and phenocopies *LIPTER*^KO^ hiPSC-CMs. **a**, RT-qPCR analysis of *cTnT, LIPTER, MYH10* and *NKX2.5* expressions in WT hiPSC-CMs after infection with AAV9 virus carrying scramble control shRNA or two NKX2.5-shRNAs. (n = 5 independent experiments). **b**, Representative images of Oil Red O lipid staining for LD accumulation, and NKX2.5, cTnT, DAPI immunostaining in WT, *LIPTER* ^KO^ and *LIPTER* ^KO/OE^ hiPSC-CMs treated with control shRNA or NKX2.5-shRNA under low (5.5 mM) or high glucose (22 mM) conditions. The experiment was carried out 4 times with similar outcomes. **c**, Quantification of the ratios of Oil Red O positive areas in cTnT^+^ CM areas in **b**. (n = 4 independent experiments). **d**, Representative immunofluorescent images of TUNEL, NKX2.5, cTnT and DAPI staining in WT, *LIPTER* ^KO^ and *LIPTER* ^KO/OE^ hiPSC-CMs treated with control or NKX2.5 shRNA under high glucose conditions (22 mM). The experiment was carried out 4 times with similar outcomes. **e**, Quantification of the ratios of TUNEL^+^ CMs in cTnT^+^ CMs of (**d**). (n = 4 independent experiments). In **a**,**c**,**e**, bars represent mean values ± s.e.m. Unpaired two-tailed *t-test* is used for comparison. ****p < 0.0001, ***p < 0.001, **p < 0.01, *p < 0.05. Source numerical data are available in source data. Source data

**Extended Data Fig. 6:**
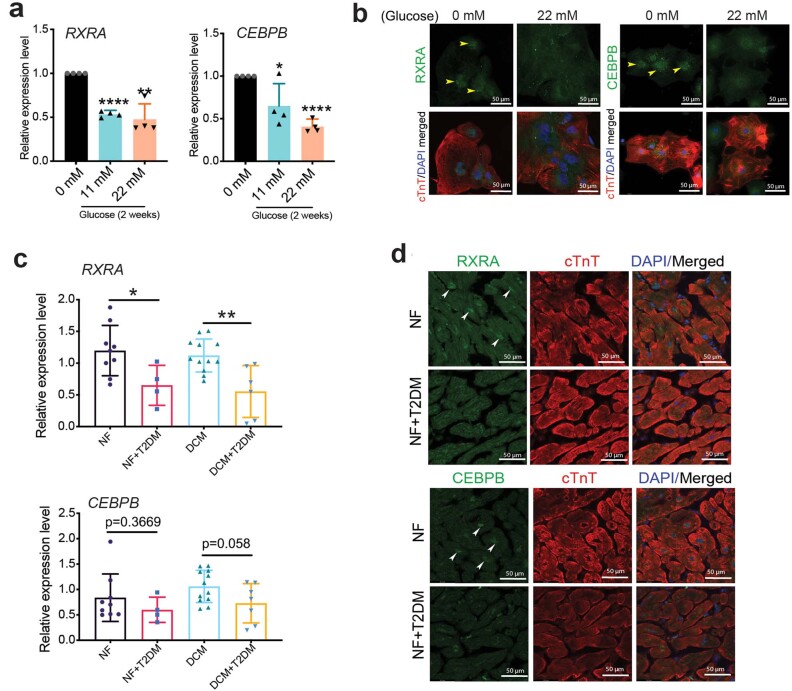
Transcriptional control of *LIPTER* in human CMs. **a**, RT-qPCR analysis of *RXRA* and *CEBPB* expressions in WT hiPSC-CMs treated with high glucose for 2 weeks. (n = 4 independent experiments). **b**, Representative immunofluorescent images of RXRA and CEBPB staining in WT hiPSC-CMs treated with or without high glucose for 2 weeks. The experiment was carried out 3 times with similar outcomes. **c**, RT-qPCR detection of *RXRA* and *CEBPB* mRNA levels in human left ventricle tissues. NF (n = 9 samples), NF+T2DM (n = 4 samples), DCM (n = 12 samples) and DCM+T2DM (n = 7 samples). **d**, Representative immunofluorescent images of RXRA and CEBPB staining in human left ventricle tissues. The experiment was carried out with 3 samples per group with similar outcomes. In **a**,**c**, bars represent mean values ± s.e.m. Unpaired two-tailed *t-test* is used for comparison. ****p < 0.0001, **p < 0.01, *p < 0.05. Source numerical data are available in source data. Source data

Since *LIPTER* expression is enriched in human CMs and downregulated in T2DM hearts (Figs. 1b,c and 4a) and extensive LDs were observed in both *LIPTER*^KO^ and T2DM human CMs (Figs. 2b,c and 4b,c), we posited that *LIPTER* transcription was under the control of a CM-specific regulatory mechanism that could respond to hyperglycaemia. The *LIPTER* promoter (~2.5 kb upstream of transcriptional start site) was predicted to contain multiple putative binding sites for *NKX2.5* *RXRA* and *CEBPB*, which are associated with CM-specific gene regulation and lipid metabolism (Fig. 4d). *NKX2.5* was the most effective in increasing *LIPTER* promotor activity, and its co-transfection with *RXRA* or *CEBPB* further enhanced the activity (Fig. 4e). Knockdown of *NKX2.5* (*NKX2.5*^KD^) by *NKX2.5*-shRNAs led to reduced expressions of *NKX2.5* and *LIPTER* (Fig. 4f), but not CM markers *cTnT* and *MYH10* (Extended Data Fig. 5a). *NKX2.5*^KD^ also prominently increased LD accumulation and apoptosis of WT hiPSC-CMs, phenocopying *LIPTER*^KO^ hiPSC-CMs (Fig. 4g,h and Extended Data Fig. 5b–e, shNKX2.5 versus shControl in WT CMs). All these results indicate that *NKX2.5* can control *LIPTER* transcription in human CMs. Next, we found exposure to high glucose levels (11.0 and 22.0 mM) for 14 days significantly reduced *LIPTER* expression (Fig. 4i), and the mRNA and nuclear protein levels of *NKX2.5*, *RXRA* and *CEBPB* in WT hiPSC-CMs (Fig. 4j,k and Extended Data Fig. 6a,b). Similar reductions in these TFs were observed in T2DM hearts compared with non-T2DM human hearts (Fig. 4l,m and Extended Data Fig. 6c,d). These results are consistent with previous reports that high glucose suppressed *NKX2.5* (ref. ^32^) and *RXRA*^33^ expressions in vivo. Finally, *LIPTER* overexpression was found to repress *NKX2.5*^KD^-induced LD accumulation and apoptosis (Extended Data Fig. 5b–e, shNKX2.5 versus shControl in *LIPTER*^KO/OE^ CMs), suggesting a therapeutic potential of *LIPTER* for treating diabetes-associated dilated cardiomyopathy (DCM)^34^. Altogether, these results demonstrate that *NKX2.5* is the primarily regulator of CM-specific *LIPTER* transcription and that hyperglycaemia can repress the *NKX2.5*–*LIPTER* axis expression in human CMs.

### *LIPTER* selectively binds phospholipids and MYH10 protein

**Fig. 5:**
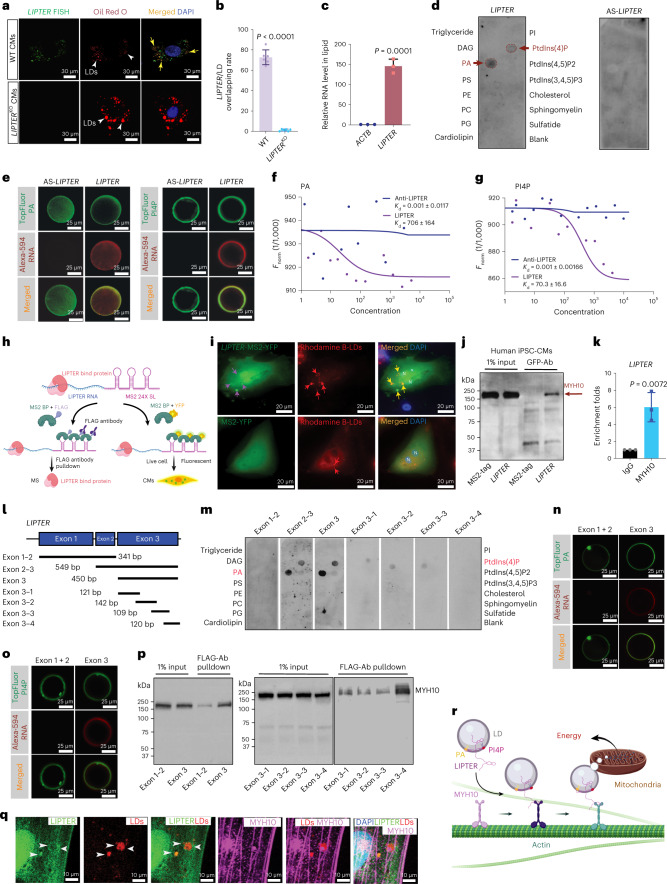
*LIPTER* selectively binds PA and PI4P on LDs and MYH10 protein. **a**, RNA FISH detecting cytosolic co-localization of *LIPTER* with LDs stained by Oil Red O staining. WT and *LIPTER*^KO^ hiPSC-CMs were treated with palmitic acid (200 μM) for 6 h to induce LD formation. **b**, Quantification of *LIPTER* co-localization with LDs in WT hiPSC-CMs (*n* = 6 independent experiments). **c**, RT–qPCR detection of *LIPTER* and *ACTB* RNA enrichments in total lipids isolated from WT hiPSC-CMs (*n* = 3 independent experiments). **d**, RNA–lipid overlay assay showing selective interaction of *LIPTER* with PA and PI4P. AS-*LIPTER* is control. PS, phosphatidylserine; PE, phosphatidylethanolamine; DAG, diacylglycerol; cholesterol; PC, phosphatidylcholine; sphingomyelin; PG, phosphatidylglycerol. **e**, Interactions between giant lipid vesicles formed with TopFluor-labelled PA/PI4P and Alexa594-labelled *LIPTER*/AS-*LIPTER*. **f**,**g**, MST quantifying PA (**f**) and PI4P (**g**) interactions with *LIPTER* or AS-*LIPTER*. **h**, Schematic of the MS2-BioTRAP system for *LIPTER* binding protein pulldown and live cell *LIPTER* tracing. **i**, Top: MS2^YFP^ protein binds MS2-tagged *LIPTER* to form particles (purple arrows), co-localizing with Rhodamine B-palmitic acid-labelled LDs (red arrows) in WT hiPSC-CMs (yellow arrows). Bottom: WT hiPSC-CMs expressing MS2^YFP^ protein and empty MS2-tag vector show evenly distributed YFP without formation of particles. **j**, Western blot showing MYH10 pulldown by anti-GFP antibody in hiPSC-CMs expressing MS2-*LIPTER*/-AS-*LIPTER* and MS2^YFP^, representative of three independent experiments. **k**, *LIPTER* enrichment by anti-MYH10 antibody in WT hiPSC-CMs (*n* = 3 independent experiments). **l**, Schematic of truncated *LIPTER* fragments. **m**, RNA–lipid overlay assay detecting interactions of truncated *LIPTER* with PA and PI4P. **n**,**o**, Images showing interactions of giant lipid vesicles formed by TopFluor-PA (**n**) and TopFluor-PI4P (**o**) with Alexa594-labelled exon 1 + 2 and exon 3 of *LIPTER*. **p**, Western blot of MYH10 pulldown in HEK293T cells transfected with MS2-tagged *LIPTER* fragments and MS2^FLAG^. **q**, Confocal fluorescence images showing co-localizations of *LIPTER-*MS2^YFP^, MYH10 and LDs in WT hiPSC-CMs. **r**, Model of human intramyocyte LD transport system via *LIPTER* and MYH10-ACTIN cytoskeleton. In **b**, **c** and **k**, bars are presented as mean ± s.e.m. Unpaired two-tailed *t*-test is used for comparison. Source numerical data and unprocessed blots are available in source data. Source data

**Extended Data Fig. 7:**
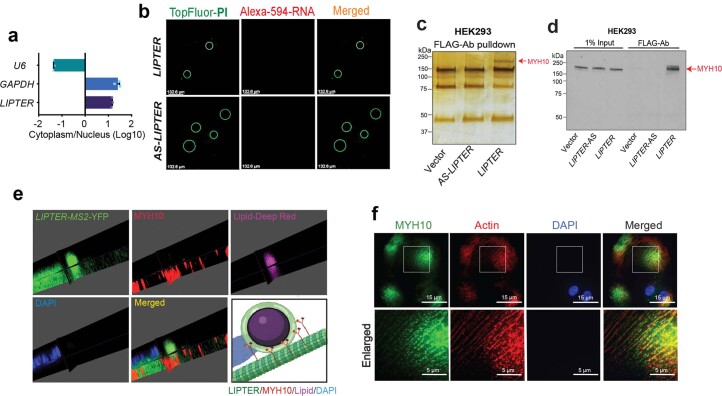
Selective binding of *LIPTER* with PA/PI4P and MYH10. **a**, RT-qPCR analysis of *LIPTER*, *U6* (nuclear RNA) and *GAPDH* (cytosolic RNA) expression levels in fractionated nuclei and cytoplasm of hiPSC-CMs. Bars represent mean values ± s.e.m. (n = 3 independent experiments). **b**, Representative images showing no detectable interactions of giant lipid vesicles generated by TopFluor-PI with Alexa594-labeled *LIPTER* or Antisense*-LIPTER* (AS-*LIPTER*). The experiment was carried out 3 times with similar outcomes. **c**, A specific protein band only pulled down by *LIPTER-*MS2 in HEK293T cells and visualized in SDS PAGE gel by silver staining. MS2 binding protein was FLAG-tagged. The experiment was carried out 2 times with similar outcomes. **d**, Western blotting confirming MYH10 pulldown by *LIPTER* in HEK293T cells. The experiment was carried out 2 times with similar outcomes. **e**, 3D confocal immunofluorescent images showing colocalization of *LIPTER*^YFP-MS2^, MYH10, and LD in WT hiPSC-CMs (also see Supplementary Video 1). The lower right graph indicates the view of merged images. The experiment was carried out 3 times with similar outcomes. **f**, Immunofluorescent images showing MYH10-ACTIN cytoskeleton in WT hiPSC-CMs. The experiment was carried out 3 times with similar outcomes. Source numerical data and unprocessed blots are available in source data. Source data

We sought to elucidate the molecular mechanisms by which *LIPTER* regulates LD transport. RT–qPCR detected cytosolic *LIPTER* expression with *GAPDH*, but not in the nucleus of hiPSC-CMs (Extended Data Fig. 7a). RNA fluorescence in situ hybridization (RNA FISH) revealed that ~74% of LDs co-localized with *LIPTER* (Fig. 5a,b), suggesting potential binding between *LIPTER* and LDs. We then isolated total lipids from WT hiPSC-CMs, which contain lipid-binding RNAs, followed with RT–qPCR. Compared with the house-keeping gene *ACTB*, *LIPTER* was highly enriched in isolated lipids (>150-fold, Fig. 5c), suggesting a possible direct interaction between *LIPTER* and lipids. Next, using the RNA–lipid overlay assay^35^, we identified that *LIPTER* selectively bound PA and PI4P, whereas antisense*-LIPTER* (AS*-LIPTER*) could not bind any lipid (Fig. 5d). Given that PA and PI4P are found on the LD membranes, we asked whether *LIPTER* could bind PA and PI4P in a membrane context. Giant unilamellar vesicles (GUVs) generated with TopFluor-labelled PI4P/PA were incubated with Alexa-594-labelled *LIPTER/*AS-*LIPTER*, respectively. *LIPTER* bound both PA- and PI4P-GUVs, whereas AS*-LIPTER* did not (Fig. 5e). Furthermore, neither *LIPTER* nor AS-*LIPTER* bound GUVs generated by PI (Extended Data Fig. 7b). The binding affinities of *LIPTER*-PA and *LIPTER*-PI4P were assessed using microscale thermophoresis (MST) assay^36^, with *K*_d_ values of 706 and 70 in the liquid phase (Fig. 5f,g), indicating strong and specific *LIPTER*-PA and *LIPTER*-PI4P interactions, respectively. Collectively, these data reveal the selective binding of *LIPTER* with PA and PI4P on LD membranes.

Next, we employed the MS2-BioTRAP system to identify *LIPTER-*interactive proteins and trace *LIPTER* in live hiPSC-CMs as previously described^37^ (Fig. 5h). As shown in Fig. 5i lower panels, control hiPSC-CMs expressed MS2-tag and MS2 binding protein MS2^YFP^, which was ubiquitously distributed in CMs. In comparison, MS2^YFP^ bound to MS2-tagged-*LIPTER*, forming green *LIPTER-*MS2^YFP^ particles observed only in the cytoplasm and co-localized with LDs (Fig. 5i, top right, yellow arrows). Since cytosolic *LIPTER*–LD co-localization was observed by both MS2-BioTRAP and RNA FISH (Fig. 5a,i), these results indicate that the MS2-tag did not affect cellular location and interaction of *LIPTER. LIPTER*-interacting proteins were then pulled down in 293T cells and hiPSC-CMs using antibody against MS2^FLAG^ or MS2^YFP^, respectively, followed by MS analysis and western blotting validation. *LIPTER* specifically pulled down non-muscle Myosin IIB (NM-IIB, or MYH10) protein, as compared with AS-*LIPTER* (Fig. 5j and Extended Data Fig. 7c,d). RNA immunoprecipitation further validated *LIPTER* enrichment by anti-MYH10 antibody in hiPSC-CMs (Fig. 5k). Confocal fluorescence microscopy revealed co-localizations of *LIPTER* with LDs and MYH10 in hiPSC-CMs (Extended Data Fig. 7e and Supplementary Video 1). Live cell imaging showed *LIPTER-*MS2^YFP^ co-localizing with migrating LDs in the cytosol of hiPSC-CMs (Supplementary Video 2). We observed the MYH10-ACTIN cytoskeleton in hiPSC-CMs (Extended Data Fig. 7f), as myosin motors move along Actin filaments^16,17^. Altogether, these results demonstrate that *LIPTER* can interact with the cytoskeleton via binding MYH10.

To identify *LIPTER* domains that specifically interact with PA, PI4P and MYH10, we generated truncated *LIPTER* fragments (Fig. 5l) for RNA–lipid overlay and GUVs assays. Exon 3 of *LIPTER* was found to contain binding domains with PA and PI4P (Fig. 5m–o). Further analysis revealed the PI4P binding domain on exon 3–1, and PA binding domains on both exon 3–2 and exon 3–3 regions (Fig. 5m). A MYH10-binding domain was identified on exon 3–4 (Fig. 5p). LD*–LIPTER*–MYH10 interactions were observed in hiPSC-CM using confocal fluorescence microscopy (Fig. 5q). These results demonstrate that *LIPTER* selectively binds PA, PI4P and MYH10 via distinct RNA domains. Overall, our data demonstrate that *LIPTER* functions as a molecular linker, connecting LDs with the cytoskeleton for intramyocyte LD transport (Fig. 5r).

### *MYH10* is indispensable for *LIPTER* function in CMs

**Fig. 6:**
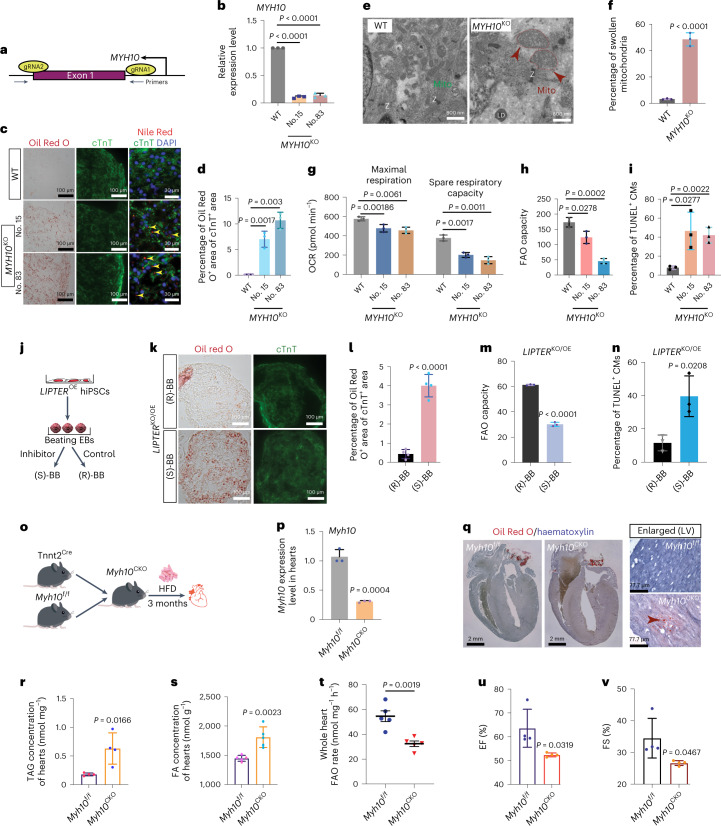
Loss-of-*MYH10* phenocopies *LIPTER* deficiency in CMs. **a**, CRISPR/Cas-9-mediated deletion of *MYH10* exon 1 in hiPSCs. **b**, RT–qPCR detection of *MYH10* expressions in WT and *MYH10*^KO^ hiPSC-CMs (*n* = 3 independent experiments). **c**, Representative images of Oil Red O staining and cTnT immunostaining (first two columns), and Nile Red for LDs and cTnT co-staining (third column) on *MYH10*^KO^ and WT hiPSC-EB sections. **d**, Quantification of Oil Red O^+^ areas in cTnT^+^ areas in **c** (*n* = 3 independent experiments). **e**, TEM showing mitochondrial morphologies in WT and *MYH10*^KO^ hiPSC-CMs. Normal (green arrows) and swollen (red arrowheads) mitochondria indicated. **f**, Quantification of swollen mitochondria ratios in **e** (*n* = 3 independent experiments). **g**,**h**, Statistical results of maximum respiratory capacity and spare respiratory capacity (*n* = 3 independent experiments) (**g**), and FAO capacities (*n* = 3 independent experiments) in WT and *MYH10*^KO^ hiPSC-CMs (**h**). **i**, Quantification of TUNEL^+^ CMs ratios in *MYH10*^KO^ and WT hiPSC-EBs (*n* = 3 independent experiments). **j**, Scheme of inhibiting MYH10 function in *LIPTER*-overexpressing hiPSC-CMs. **k**, Representative images of Oil Red O staining and cTnT immunostaining in *LIPTER*^KO/OE^ hiPSC-CMs after treatment with (S)-(−)-Blebbistatin ((S)-BB) or control (R)-(−)-Blebbistatin ((R)-BB) for 10 days. **l**, Ratios of Oil Red O^+^ areas in cTnT^+^ CM areas in **k** (*n* = 4 independent experiments). **m**, FAO capacities of *LIPTER*^KO/OE^ hiPSC-CMs after treating with (S)-BB or (R)-BB for 10 days (*n* = 3 independent experiments). **n**, Ratios of TUNEL^+^ CM in *LIPTER*^KO/OE^ hiPSC-CMs after same treatment in **m** (*n* = 3 independent experiments). **o**, Scheme for HFD feeding of mice with conditional *Myh10* knockout in CMs. **p**, RT–qPCR detecting *Myh10* expression levels in mouse hearts. **q**–**s**, Representative images of Oil Red O lipid staining of mouse hearts (**q**), and quantifications of TAG (**r**) and FA (**s**) concentrations in mouse hearts after 3 months of HFD feeding (*n* = 4 mice). **t**, FAO rates of whole *Myh10*^f/f^ and *Myh10*^CKO^ mouse hearts post HDF feeding (*n* = 5 mice). **u**,**v**, Cardiac EF (**u**) and FS (**v**) measurements of *Myh10*^f/f^ and *Myh10*^CKO^ mice post HFD feeding (*n* = 4 mice). In **b**, **f**, **d**–**i**, **l**–**n** and **r**–**v**, bars are presented as mean ± s.e.m. Unpaired two-tailed *t*-test is used for comparison. Source numerical data are available in source data. Source data

**Extended Data Fig. 8:**
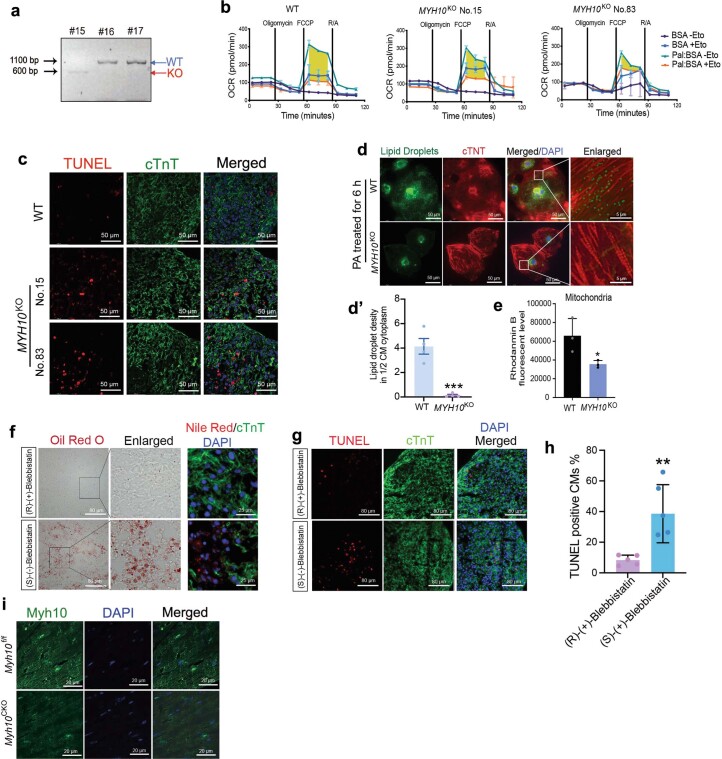
*MYH10* deficiency phenocopies *LIPTER*^KO^ in CMs. **a**, PCR detection of *MYH10* null hiPSC clones confirms successful knockout of *MYH10*. **b**, Mitochondria stress assay results collected from a Seahorse XF96 Analyzer showing the differences of maximal oxygen consumption values with Palmitate:BSA between conditions without and with Etomoxir (Eto). The yellow zone in panels represent FAO capabilities of WT and *MYH10*^KO^ hiPSC-CMs. **c**, Representative immunofluorescent images for TUNEL and cTnT co-staining in WT and *MYH10* ^KO^ hiPSC-EB sections. **d**, Representative images showing LD accumulation and cytosolic distribution in WT and *MYH10*^KO^ hiPSC-CMs treated with 200 μM palmitic acid for 6 h. The experiment was carried out 4 times with similar outcomes. **d'**, Quantification of relative LD density in the 1/2 cytosolic areas of CMs in **d**. (n = 4 independent experiments). **e**, Quantification of fluorescence levels in mitochondria isolated from WT and *MYH10*^KO^ hiPSC-CMs after Rhodamine B-palmitic acid treatment for 2 h. (n = 3 independent experiments). **f**, Representative images of Oil Red O lipid staining (first two columns); Nile Red and cTnT co-staining (3 rd column) in WT hiPSC-EBs treated with (S)-(-)-Blebbistatin or (R)-(-)-Blebbistatin for 10 days. **g**, Representative images of TUNEL and cTnT co-staining in WT hiPSC-EBs treated with (S)-(-)-Blebbistatin or (R)-(-)-Blebbistatin for 10 days. **h**, Quantification of TUNEL^+^ CM ratios in WT hiPSC-CMs treated with (S)-(-)-Blebbistatin or (R)-(-)-Blebbistatin for 10 days. (n = 5 independent experiments). **i**, Immunofluorescent images showing Myh10 deficiency in *Myh10*^CKO^ mouse heart. In **d'**, **e**, **h**, bars represent mean values ± s.e.m. Unpaired two-tailed *t-test* is used for comparison. ***p < 0.001, **p < 0.01, *p < 0.05. Source numerical data are available in source data. Source data

*MYH10* was knocked out (*MYH10*^KO^) in hiPSCs using CRISPR/Cas-9 (Fig. 6a,b and Extended Data Fig. 8a). Compared with WT hiPSC-CMs, *MYH10*^KO^ hiPSC-CMs displayed increased LD accumulation (Fig. 6c,d), ~50% swollen mitochondria (Fig. 6e,f), reduced mitochondrial spare respiratory and maximal respiration capacities (Fig. 6g), and decreased FAO capacity (Fig. 6h and Extended Data Fig. 8b). Additionally, *MYH10*^KO^ hiPSC-CMs exhibited increased apoptosis (Fig. 6i and Extended Data Fig. 8c), retarded LD transport (Extended Data Fig. 8d,d′), reduced LD–mitochondria fusion rate (third row, Extended Data Fig. 4e,f) and decreased Rhodamine-B-labelled palmitic acid transport into mitochondria (Extended Data Fig. 8e). Additionally, inhibition of MYH10 ATPase activity with (*S*)-(−)-Blebbistatin^38^ led to enhanced LD accumulation and apoptosis in WT hiPSC-CMs compared with its inactive enantiomer control, (*R*)-(+)-Blebbistatin (Extended Data Fig. 8f–h). These results demonstrate that loss-of-MYH10 phenocopies *LIPTER* deficiency in hiPSC-CMs. We then investigated whether MYH10 is required for *LIPTER* function by inhibiting MYH10 in *LIPTER*-overexpressing CMs (Fig. 6j). Compared with (*R*)-(+)-Blebbistatin, (*S*)-(−)-Blebbistatin treatment enhanced LD accumulation (Fig. 6k,l), reduced FAO capacity (Fig. 6m) and elevated apoptosis (Fig. 6n) in *LIPTER*^KO/OE^ hiPSC-CMs. These results reveal that inhibition of MYH10 could abolish *LIPTER* gain-of-function, indicating the critical role of MYH10 in executing *LIPTER* function.

Unlike *LIPTER*, *MYH10* is conserved in humans and mice. Therefore, *Myh10* was conditionally knocked out in mouse CMs (*Myh10*^CKO^) by crossing *Myh10*^f/f^ with Tnnt2^Cre^ mice (Fig. 6o–p and Extended Data Fig. 8i). Fed with high-fat diet (HFD, 45 kcal% fat) for 3 months, *Myh10*^CKO^ male mice exhibited severe cardiac lipid accumulation, including enhanced LD deposition (Fig. 6q, arrowhead) and elevated concentrations of TAG and FAs, compared with *Myh10*^f/f^ littermates (Fig. 6r,s). Notably, *Myh10* deficiency significantly reduced the FAO rates of whole *Myh10*^CKO^ hearts compared with *Myh10*^f/f^ hearts (Fig. 6t). Lastly, left ventricular ejection fraction (EF) and fractional shortening (FS) values significantly declined in *Myh10*^CKO^ mice compared with *Myh10*^f/f^ mice (Fig. 6u,v). Collectively, all these in vitro and in vivo results demonstrate a conserved and critical role of *Myh10* in LD metabolism of mammalian heart muscle cells.

### Gain-of-*LIPTER* mitigates lipotoxicity of hiPSC-CMs

**Fig. 7:**
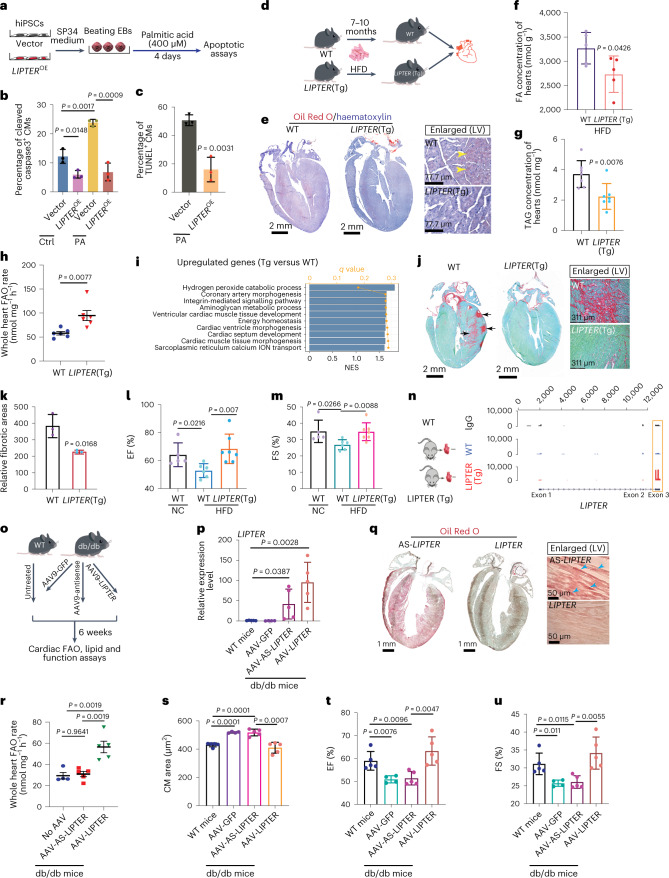
*LIPTER* transgene mitigates cardiomyopathies and cardiac dysfunctions in HFD-fed and *Lepr*^db/db^ mice. **a**, Scheme of CM lipotoxicity assay with 400 μM palmitic acid treatment on hiPSC-CMs for 4 days. **b**,**c**, Cleaved caspase3^+^ CM ratios (**b**) and TUNEL^+^ CM ratios (**c**) in control and *LIPTER*^OE^ hiPSC-CMs after PA treatment (**b**,**c**, *n* = 3 independent experiments). **d**, Schematic of investigating *LIPTER*(Tg) effects on cardiac abnormalities in HFD‐fed mice. **e**, Oil Red O and haematoxylin staining of mouse hearts after 7 month HFD feeding. **f**,**g**, FA (**f**, *n* = 5 mice each group) and TAG (**g**, *n* = 7 mice each group) concentrations were next measured. **h**, FAO rates of whole WT and *LIPTER*(Tg) mouse hearts at 3 months of age (*n* = 5 mice each group). **i**, GO biological processes significantly enriched in the upregulated genes in *LIPTER* (Tg) versus WT mouse hearts. **j**, Picrosirius Red and Fast Green co-staining of mouse hearts after 10 month HFD feeding. **k**, Quantification of relative red fibrotic areas in whole hearts (*n* = 3 mice each group). **l**,**m**, EF (**l**) and FS (**m**) measurements of WT and *LIPTER*(Tg) mice fed on HFD or NC for 10 months. *n* = 5 mice (WT + NC), *n* = 6 mice (WT + HFD), *n* = 7 mice (Tg + HFD). **n**, RIP-seq reads plot showing *LIPTER* pulldown by anti-Myh10 antibody in *LIPTER* (Tg) mouse heart. Red arrow indicates the reads enrichment on *LIPTER* exon 3. The experiment was carried out in two mice, each genotype with similar outcomes. **o**, A scheme for delivering *LIPTER* and controls (GFP, AS-*LIPTER*) into *Lepr*^db/db^ mouse CMs using AAV9 virus. **p**, RT–qPCR detecting *LIPTER* expressions in *Lepr*^db/db^ mouse hearts 6 weeks post AAV9 injection (*n* = 5 mice per group, except *n* = 4 mice in the GFP group). **q**, Oil Red O staining of *Lepr*^db/db^ mouse hearts 6 weeks post AAV9 injection. **r**, FAO rates of *Lepr*^db/db^ mouse hearts 6 weeks post AAV9 injection (*n* = 5 mice per group, except *n* = 4 mice in the No AAV group). **s**, CM size analysis using WGA staining 6 weeks post AAV9 injection (*n* = 5 mice per group, except *n* = 4 mice in the GFP group). **t**,**u**, EF (**t**) and FS (**u**) measurements in WT and *Lepr*^db/db^ mice 6 weeks post AAV9 injection (*n* = 5 mice per group, except *n* = 4 mice in the GFP group). In **b**, **c**, **f**–**h**, **k**–**m**, **p** and **r**–**u**, bars are presented as mean ± s.e.m. Unpaired two-tailed *t*-test is used for comparison. Source numerical data are available in source data. Source data

**Extended Data Fig. 9:**
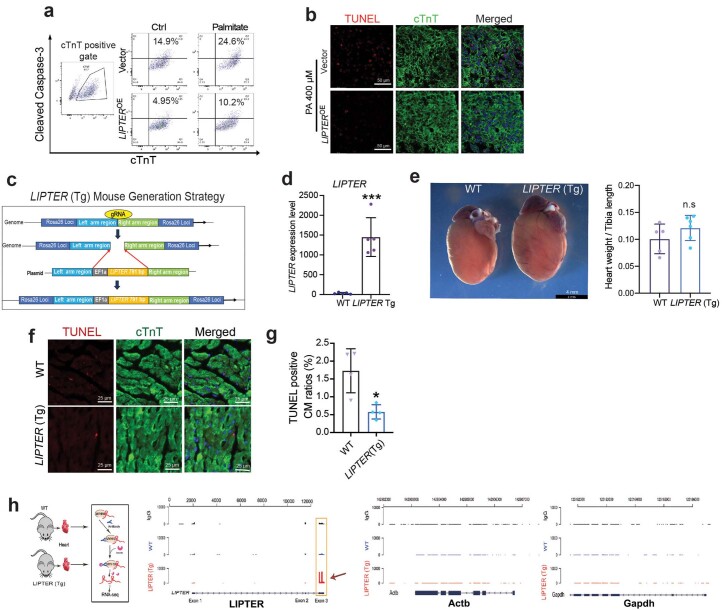
*LIPTER* transgene displays cardiac protective effects in HFD-fed mice. **a**, Representative FACS results showing cleaved-Caspase-3/cTnT double positive cell ratios in control and *LIPTER*^OE^ hiPSC-CMs treated with or without 400 μM palmitic acid for 4 days. The experiment was carried out 3 times with similar outcomes. **b**, Representative Immunofluorescent images of TUNEL and cTnT co-staining in control and *LIPTER*^OE^ hiPSC-EBs treated with 400 μM palmitic acid for 4 days. The experiment was carried out 3 times with similar outcomes. **c**, Schematic of *LIPTER*(Tg) mouse generation using CRISPR/Cas-9, with the Rosa26 locus targeted by a single gRNA. **d**, RT-qPCR examining *LIPTER* expression levels in WT and *LIPTER*(Tg) mouse hearts. (n = 5 mice per group). **e**, Representative images of WT and *LIPTER* (Tg) mouse hearts after 7 months of HFD feeding. The heart weight/tibia length ratio was calculated. (n = 5 mice in WT, and n = 6 in Tg). **f**, Representative immunofluorescent images of TUNEL and cTnT co-staining in mouse heart sections after 7 months of HFD feeding. The experiment was carried out 4 times with similar outcomes. **g**, Quantification of TUNEL^+^ CM ratios in (**f**). (n = 4 mice per group). **h**, RIP-seq results showing *LIPTER* pulldown in *LIPTER* (Tg) mouse heart using anti-Myh10 antibody, with the red arrow indicating enriched signals on exon 3 of transgenic *LIPTER*. Control *Actb* and *Gapdh* RNAs were not pulled down by anti-Myh10 antibody. The experiment was carried out in 2 mice each genotype with similar outcomes. In **d**, **e**, **g**, bars represent mean values ± s.e.m. Unpaired two-tailed *t-test* is used for comparison. ***p < 0.001, *p < 0.05, (n.s), no significance. Source numerical data are available in source data. Source data

Palmitate overload has been shown to induce cardiac lipotoxicity in vivo, which compromised mouse heart function and increased CM death^39^. In vitro, palmitate overload can incite oxidative and ER stress of CMs^2^. We found that palmitic acid (400 µM) treatment for 4 days significantly induced apoptosis in hiPSC-CMs (Fig. 7a,b and Extended Data Fig. 9a, Vector-Ctrl versus Vector-PA), which was prominently reduced by *LIPTER*^OE^ (Fig. 7b,c, *LIPTER*^OE^-PA versus Vector-PA; Extended Data Fig. 9a,b). These data reveal an in vitro protective role of *LIPTER* against lipotoxicity.

### In vivo *LIPTER* transgene displays a cardiac protective role

Intramyocyte LD accumulation occurs in human obese and diabetic hearts when cardiac function is still normal^5,39^, suggesting that LD metabolic disturbance precedes the onset of cardiac dysfunction. We hypothesized that gain-of-*LIPTER* function could alleviate obesity- and diabetes-associated cardiomyopathies and cardiac dysfunctions. To test this, we generated a *LIPTER* transgenic (*LIPTER*(Tg)) mouse line by knocking in *LIPTER* into the Rosa26 locus, resulting in high *LIPTER* expression in the heart (Extended Data Fig. 9c,d). Six-week-old male *LIPTER*(Tg) and WT mice were fed a HFD (45 kcal% fat) to induce obesity, insulin resistance and cardiac abnormalities as previously reported (Fig. 7d)^40,41^. After 7 months, HFD-fed *LIPTER*(Tg) mice showed no significant change in heart weight/tibia length ratios compared with WT mice (Extended Data Fig. 9e). However, *LIPTER*(Tg) significantly reduced LD levels, and FA and TAG concentrations compared with WT hearts (Fig. 7e–g). Notably, *LIPTER*(Tg) mice exhibited significantly enhanced FAO rates in whole hearts (Fig. 7h) and globally altered transcriptome (Supplementary Table 4). Metabolic process, energy homeostasis, cardiac muscle development and growth, and sarcoplasmic reticulum Ca^2+^ transport biological processes were significantly over-represented in the *LIPTER*(Tg) upregulated genes (Fig. 7i). We also observed significantly reduced fibrotic areas (Fig. 7j,k) and apoptosis (Extended Data Fig. 9f,g) in HFD-fed *LIPTER*(Tg) hearts compared with similarly treated WT hearts. Importantly, *LIPTER*(Tg) preserved cardiac function in HFD-fed mice. Significantly reduced EF and FS values were observed in WT mice fed with HFD for 10 months compared with WT mice fed with normal chew (NC) (Fig. 7l,m, WT-HDF versus WT-NC). However, EF/FS values of HFD-fed *LIPTER*(Tg) mice were significantly higher than those of HFD-fed WT mice and were preserved at levels similar to NC-fed WT mice (Fig. 7l,m, *LIPTER*(Tg)-HFD versus WT-HFD). These results demonstrate that gain-of-*LIPTER* can mitigate HFD-induced cardiomyopathy and preserve cardiac function. Furthermore, to test whether the transgenic *LIPTER* could interact with mouse Myh10 protein, we performed RNA immunoprecipitation sequencing (RIP-seq) to pull down and sequence all mouse Myh10-binding RNAs from *LIPTER*(Tg) and WT mouse hearts. Although no Myh10 binding signals were detected on control *Actb* and *Gapdh* mRNAs, mouse Myh10 protein bound *LIPTER* in *LIPTER*(Tg) heart (Extended Data Fig. 9h). Interestingly, mouse Myh10 binding signals were enriched on exon 3 of transgenic *LIPTER* (Fig. 7n, red arrow), which was found to interact with human MYH10 (Fig. 5p). These results suggest that *LIPTER*–MYH10 interaction might play a conserved role in LD transport of mammalian CMs, although the mouse homologue of *LIPTER* remains to be identified.

**Extended Data Fig. 10:**
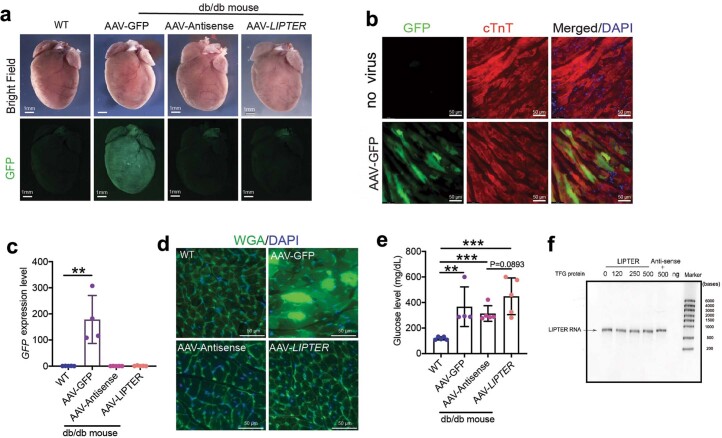
CM-targeted *LIPTER* transgene exhibits cardiac protective effects in *Lepr*^db/db^ mice. **a**, Heart images of WT and db/db mice 6 weeks after injection of AAV9-cTnT-*GFP*, AAV9-cTnT-AS-*LIPTER* or AAV9-cTnT-*LIPTER*, with fluorescent images showing robust GFP expression post AAV9-cTnT-GFP injection. The experiment was carried out 4 times with similar outcomes. **b**, Representative images showing GFP expression in CMs of db/db mouse heart 6 weeks after AAV9-cTnT-GFP injection. The experiment was carried out 4 times with similar outcomes. **c**, RT-qPCR examining GFP expression levels in WT and db/db mouse hearts 6 weeks after AAV9 injection. (n = 5 mice per group, except n = 4 mice in the GFP group). **d**, Representative images of WGA staining in WT or db/db mouse hearts 6 weeks after AAV9 injection. The experiment was carried out 5 times with similar outcomes. **e**, Blood glucose levels of WT and db/db mice 6 weeks after AAV9 injection. WT mice without AAV9 injection also serve as control. (n = 5 mice per group, except n = 4 mice in the GFP group). **f**, EMSA results showing no direct interaction between *LIPTER* and TFG protein. The experiment was carried out 2 times with similar outcomes. In **c** and **e**, bars represent mean values ± s.e.m. Unpaired two-tailed *t-test* is used for comparison. ***p < 0.001, **p < 0.01. Source numerical data are available in source data. Source data

Finally, we examined the impact of CM-specific *LIPTER* transgene on cardiomyopathy of leptin-receptor-deficient *Lepr*^db/db^ mouse (Fig. 7o), a model of T2DM and obesity that develops cardiac hypertrophy and dysfunction from 10 weeks of age^42,43^. We employed an AAV9 virus carrying a chicken *cTnT* promoter to selectively deliver *LIPTER* into CMs^44^. Retro-orbital injection of AAV9-cTnT-GFP virus (2 × 10^10^ vg/g) into *Lepr*^db/db^ mice led to robust GFP expression in mouse CMs (Extended Data Fig. 10a,b). Subsequently, AAV9-*LIPTER*, control AAV9-GFP and AAV9-AS-*LIPTER* viruses were administered to 6-week-old male B6.BKS(D)-*Lepr*^db/db^ mice using the same approach. Age-matched WT C57BL/6J male mice without AAV-9 injection also served as controls. Six weeks post AAV-9 injection, transgenic *LIPTER*, AS-*LIPTER* and GFP expressions were detected in the mouse hearts (Fig. 7p and Extended Data Fig. 10c). AAV9-*LIPTER* administration resulted in reduced intramyocyte LD accumulation (Fig. 7q), increased whole heart FAO rates (Fig. 7r), reduced CM size (Fig. 7s and Extended Data Fig. 10d), and preserved EF and FS values in *Lepr*^db/db^ mice (Fig. 7t–u) compared with control groups. Since AAV9-*LIPTER* transgene in CMs did not impact blood glucose levels of *Lepr*^db/db^ mice (Extended Data Fig. 10e), these findings indicate that CM-restricted *LIPTER* transgene can mitigate cardiomyopathy and preserve cardiac function in *Lepr*^db/db^ mice.

## Discussion

Our data demonstrate that *LIPTER* functions as an RNA linker, facilitating LD transport in human CMs by connecting LDs via binding PA/PI4P and the cytoskeleton via binding MYH10. Despite the importance of lncRNAs in regulating cellular processes, the roles of lncRNA–lipid interactions remain largely unexplored. Lin et al. reported that *LINK-A* bound PI(3,4,5)P3 to hyperactivate AKT in cancer cells^35^, and SNHG9 was found to bind PA to facilitate LATS1 liquid–liquid phase separation, promoting oncogenic YAP signalling^45^. Our study reveals that *LIPTER* binds both PA and PI4P (Fig. 5d,e), which play crucial roles in membrane–membrane interactions that facilitate LD transfer. Specifically, PA on the ER membrane contributes to the formation of newly formed LDs^46^, while PI4P on the Golgi and plasma membrane can recruit and bind lipid transport carrier proteins on LDs^47^. Our MS analysis of proteins pulled down by *LIPTER* (Supplementary Table 5) did not detect typical LD-associated proteins, except TFG (Trafficking From ER To Golgi Regulator). However, we did not detect any interaction between TFG and *LIPTER* (Extended Data Fig. 10f). These findings, in conjunction with the GUV results (Fig. 5e), indicate that *LIPTER* can directly bind PA and PI4P on LDs without additional protein co-factors. Currently, factors determining the selective interactions of RNA with different lipids are not well understood. A few studies have suggested that RNA–lipid interaction may depend on RNA length, base pairing and nucleotide content^48,49^. Tomasz et al. also reported that guanine and G-quadruplex formation are critical for RNA–lipid interactions^49^.

Our study demonstrates that *MYH10* depletions disrupted LD metabolism and transport in hiPSC-CMs, and compromised cardiac function in *Myh10*^CKO^ mice, highlighting the crucial and conserved role of cytoskeleton in LD trafficking within mammalian CMs. Additionally, we observed enlarged LDs in *LIPTER*^KO^ hiPSC-CMs compared with WT hiPSC-CMs (Fig. 2g). *Myh9* depletion in U2OS cells was found to reduce LD dissociation from the ER and increase LD size^18^. Since *MYH9* is not expressed in human CMs, our data suggest that disconnection of MYH10 from LDs due to *LIPTER* deficiency may impair the dissociation of newly formed LDs from ER, leading to increased LD size.

Diabetic cardiomyopathy is recognized as a non-ischaemic form of DCM, characterized by progressive muscle loss, global systolic dysfunction and heart failure. It has been reported that approximately 75% of patients with idiopathic DCM are diabetic^34^. An 85-fold increase in apoptosis and 4-fold increase in necrosis of myocytes have been observed in diabetic hearts compared with non-diseased hearts^50^. Our data reveal that hyperglycaemia suppresses the *NKX2.5*–*LIPTER* axis in hiPSC-CMs, and *NKX2.5*/*LIPTER* deficiencies induce LD deposition and CM death (Extended Data Fig. 5). Although the mechanism of *NKX2.5* downregulation by hyperglycaemia remains to be investigated, our data suggest that a declined *NKX2.5*–*LIPTER* axis could possibility contribute to CM loss in diabetic hearts. We hypothesize that hyperglycaemia could differentially repress *NKX2.5*–*LIPTER* expression levels in individual CMs, and CMs with critically low *LIPTER* expression levels could gradually die, with this slow progress of muscle loss eventually leading to DCM of diabetic hearts. Since intramyocyte LD accumulation precedes onset of cardiac dysfunctions in individuals with obesity or diabetes^5,39^, our in vivo *LIPTER* transgene data strongly suggest that targeted *LIPTER* delivery into heart muscle might be an effective strategy to prevent cardiac dysfunction and heart failure in metabolic syndromes.

Compared with DNA, RNA exhibits a broader range of interacts with various molecules, suggesting more diverse and complex functional dimensions. LDs are ancient organelles that exist in prokaryotes and eukaryotic cells. Our findings reveal a molecular linker role of RNA in mediating the transport of lipid-covered vesicle through RNA–lipid/protein interactions. This may reflect ancient RNA functions in the primordial ‘RNA world’, where RNA–lipid/protein interactions could have play critical roles in forming the first living system, which encompassed lipid membranes and a mixture of RNA, protein and other molecules.

In summary, this study uncovers an lncRNA-mediated LD transport system in human CMs and reveals the crucial role of *LIPTER* and MYH10 in lipid metabolism of mammalian hearts. The mouse homologue of *LIPTER* has not been identified, limiting the in vivo loss-of-function analysis of *LIPTER* in rodents. Moreover, considering that a single lncRNA can interact with multiple proteins^28,51,52^, it is not yet clear whether *LIPTER* could regulate LD metabolism through other mechanisms, such as epigenetic regulation of genes in TAG synthesis and lipolysis on LDs (Extended Data Fig. 4a). Lastly, FAO rate measurements were conducted at a 6:1 palmitate-to-albumin ratio, much higher than the physiological ratio (1:1–3:1) (ref. ^53^), raising a concern of lipid overload during measurement. Additionally, whole heart FAO rate measurement using heart tissue homogenate were not performed under the same oxygen concentration as in vivo conditions, resulting in the majority of radiolabelled palmitate being converted into ASMs rather than CO_2_. To address these technical challenges, FAO rates will be further evaluated in live animals as previously described^54^.

## Methods

### Experimental model and subject details

#### hPSCs

WT hiPSCs were generated from fibroblasts of a healthy donor as previously in this report^55^ and transferred with an MTA. The hiPSCs were cultured on irradiated mouse embryonic fibroblast cells and maintained in KSR medium containing Dulbecco’s modified Eagle medium (DMEM)/F12 (Gibco, 11330032), 20% KSR (Invitrogen, 10828028), penicillin/streptomycin (P/S; Gibco, 15140163), GlutaMAX Supplement (Gibco, 35050061), non-essential amino acids (Gibco, 11140050), β-mercaptoethanol (Sigma-Aldrich, M3148) and bFGF (R&D Systems, 233-FB-025). For differentiation into CMs as previously described^23,56^, hiPSC-derived EBs were suspended in StemPro-34 medium (Gibco, 10639011) and treated with the following conditions: days 0–1, BMP4 (2.5 ng ml^−1^); days 1–4, BMP4 (10 ng ml^−1^), bFGF (5 ng ml^−1^) and Activin A (2 ng ml^−1^); and days 4–8, XAV-939 (Tocris, cat. no. 3748). After 20 days, beating EBs were cultured with DMEM high-glucose medium (Gibco, 11966025) containing 10% foetal bovine serum (FBS) and P/S. For monolayer CM differentiation, hiPSCs were seeded on Matrigel and treated with 12.5 nM CHIR99021 (Sigma, SML1046) in differentiation medium (RPMI-1640 with B27 minus insulin) for 24 h, which were then replaced with differentiation medium without additional factors. Two days later, cells were treated with 5 nM IWP4 (Tocris, 5214) for 48 h, and then the medium was changed every two days until day 14. The beating cells were then selected in DMEM medium containing no glucose with 4 mM lactate for four additional days. After CM selection, CMs were cultured in DMEM high-glucose medium containing 10% FBS and P/S.

#### Animals and diets

All animal experiments were performed following the approval and guidelines of the Institutional Animal Care and Use Committee of Indiana University. Animals are maintained in accordance with the applicable portions of the Animal Welfare act and the DHHS Guide for the Care and Use of Laboratory Animals. *LIPTER*(Tg) C57BL/6J mice were generated using CRISPR/Cas9 at the Genome Editing, Transgenic, and Virus Core at UPMC Magee-Women’s Research Institute. Six-week-old male homozygous *Lepr*^db/db^ mice were obtained from the Jackson Laboratory (strain number 000697). *Myh10* floxed mice with C57BL/6J background were obtained from Dr Robert S. Adelstein lab at Laboratory of Molecular Cardiology, National Heart, Lung and Blood Institute^57^. C57BL/6J *Tnnt2*^Cre^ mice were obtained from Dr Chenleng Cai lab at Indiana University. Conditional *Myh10*^cKO^ in mouse CMs was generated by crossing *Myh10*^f/f^ mice with Tnnt2^MerCreMer^ mice. Six-week-old male *Myh10*^f/f^ and *Myh10*^f/f^/*Tnnt2*^MerCreMer^ mice were injected with tamoxifen (0.1 mg g^−1^ body weight) for days 1, 3 and 5 by intraperitoneal injection and then fed with a HFD (45 kcal% fat, Research Diets Inc., D12451i) for 3 months. Six-week-old male *LIPTER*(Tg) mice and age-matched WT C57BL/6J mice were fed with a HFD (45 kcal% fat, Research Diets Inc., D12451i) or NC (18 protein, Inotiv, 2018sx) for 10 months.

### Method details

#### Human heart tissue acquisition

Human left ventricle tissues were obtained from the Duke Human Heart Repository, which is a Duke University Health System institutional review board-approved tissue repository. Samples were procured by the Duke Human Heart Repository in accordance with an approved Duke University Health System institutional review board protocol with written informed consent. All patient information was de-identified. Left ventricle tissues were immediately flash frozen in liquid nitrogen and stored in a −80 °C freezer.

### CRISPR/Cas-9 knockout

*LIPTER* and *MYH10* knockout hiPSC lines were established using CRISPR/Cas9 as we previously described^28^. The guide RNAs (gRNAs) targeting the *LIPTER* or *MYH10* gene were designed using the CRISPR design platform (https://zlab.bio/guide-design-resources), and the dual gRNA knock-out method was used. The gRNA was cloned into the pENTR-spCAS9-T2A-EGFP vector, and CRISPR/Cas-9 and gRNA plasmids were co-transfected into human iPSCs using X-tremeGENE 9 DNA Transfection Reagent (Roche, 6365787001). After transfection, GFP^+^ cells were sorted by FACSAria II cell sorter (BD Biosciences) and seeded on feeder cells with Y-27632 (Selleckchem, S1049). Single hiPSC clones were picked and genotyped by PCR.

### Plasmid construction

*LIPTER* transcripts were amplified from CMs and cloned into the pHAGE-puro-inducible vector for generating *LIPTER* overexpression vectors. The *LIPTER* promoter region (hg38, chr3:157087815-157090367) was amplified and cloned into the pGL3-basic vector. The pBABE-puro human RXR alpha plasmid and pBABE-puro LAP2 (C/EBP beta isoform) plasmid were obtained from Addgene (plasmid numbers 11441 and 15712). *NKX2.5* was cloned into the pMXs-puro plasmid. FLAG-tagged ORF1, ORF2 and ORF3 of *LIPTER* were cloned into a pQCXIP vector.

### *LIPTER* coding potential prediction and verification

Protein coding potential was predicted using Open Reading Frame Finder (https://www.ncbi.nlm.nih.gov/orffinder/). FLAG sequences (GAC TAC AAA GAC GAT GAC GAC AAG) were inserted after each predicted ORF before the stop codon and cloned into a pQCXIP vector. Three *LIPTER* ORFs with FLAG plasmids were transfected into HEK293T cells (CRL-3216, ATCC) respectively using the X-tremeGENE 9 DNA Transfection Reagent (Roche, 6365787001). A non-relevant protein-coding gene with a FLAG-tag was transfected as a positive control. RNA expression levels were detected using RT–qPCR. FLAG was detected using anti-FLAG antibody (1:1,000, Cell Signaling, 14793S) by immune fluorescent staining and western blotting.

### Luciferase assay

Luciferase assay was performed using the Dual-Glo Luciferase Assay System (Promega, E2920) following the manufacturer’s protocol. Briefly, HEK293T cells were co-transfected with the pGL3-*LIPTER*-promoter and *NKX2.5*, *CEBPB* or *RXRa* plasmids, or a vector control using the X-tremeGENE 9 transfection reagent (Roche, 6365787001). Cells were collected 48 h post-transfection. Firefly and Renilla luciferase activities in cell lysates were measured using the GLOMAX Explorer microplate reader system (Promega).

### Lipid uptake assay

Rhodamine-B-labelled palmitic acid (Avanti, 810104) was dissolved in ethanol and then in 10% bovine serum albumin (BSA). The final concentration of PA was 5 mM. HiPSC-CMs were dissociated into single cells and seeded at a density of 1 × 10^6^ cells per well in a 12-well plate. Cells were treated with 200 μM Rhodamine-B-labelled palmitic acid for 1 h and lysed with 100 μl RIPA lysis buffer. For the mitochondrial lipid uptake assay, hiPSC-CMs were treated with 200 μM Rhodamine-labelled palmitic acid for 2 h. Subsequently, mitochondria were isolated using the Mitochondria Isolation Kit for Cultured Cells (ThermoFisher, 89874) and lysed with 100 μl RIPA lysis buffer. Fluorescence values were detected at an excitation of 520 nm with a 580–640 nm filter on a GLOMAX Explorer microplate reader system (Promega).

### Mitochondrial proteomics

WT and *LIPTER*^KO^ hiPSC-CMs were selected and dissociated into single cells. Mitochondria were isolated from 5 × 10^6^ CMs using Mitochondria Isolation Kit for Cultured Cells (ThermoFisher, 89874). MS and data analyses were performed at the Proteomics Core Facility at the Indiana University School of Medicine.

### Flow cytometry

Flow cytometry was performed to detect cTnT positive cell ratios during EB differentiation, as previously described^28^. Briefly, day 20 or 40 EBs were collected and dissociated with 1 mg ml^−1^ Collagenase B for 30 min, followed by 0.25% trypsin–EDTA for 5 min at 37 °C. Dissociated single cells were fixed in 4% paraformaldehyde (PFA) for 15 minutes at room temperature and washed three times with phosphate-buffered saline (PBS). Cells were incubated in a blocking buffer containing 1× PBS, 10% goat serum and 0.1% saponin. Cells were then incubated with primary antibody diluted in 1× PBS with 2% BSA and 0.1% saponin for 1 h at 37 °C, followed by incubation with Alexa Fluor 488-labelled goat anti-mouse secondary antibody (1:200, Thermo Fisher Scientific, A-11001) for 1 h at 37 °C. Flow cytometry analysis was conducted using an Attune NxT flow cytometer (Invitrogen). Data were analysed using FlowJo (Treestar). Supplementary Fig. 1 shows the gate setting.

### Nuclear and cytoplasmic RNA detection

A total of 2 × 10^6^ hiPSC-CMs were separated into nuclear and cytoplasmic fractions using NE-PER Nuclear and Cytoplasmic Extraction Reagents (ThermoFisher, 78833). RNAs from both nuclear and cytoplasmic fractions were extracted using the RNeasy Mini Kit (Qiagen, 74106). *LIPTER*, *GAPDH* (cytosolic marker) and *U6* (nuclear marker) expression levels were detected using RT–qPCR on a QuantStudio 6 Flex system (Applied Biosystems).

### Extraction of lipid-associating RNAs

A total of 1 × 10^6^ hiPSC-CMs were treated with 100 μM palmitate acid for 48 h, followed by lipid extraction using the Lipid Extraction Kit (Cell Biolabs, STA-612) according to the manufacturer’s protocol. The isolated total lipids were dried in a vacuum concentrator at 4 °C and resuspended in 700 μl of TRIzol. RNA was then extracted by using the RNeasy mini kit (Qiagen, 74106).

### TEM

hiPSC-derived EBs at day 40 of differentiation were fixed in 2.5% glutaraldehyde in 1× PBS for 1 h. Then EBs were washed with PBS and post-fixed with 1% osmium tetroxide and 1% potassium ferricyanide for 1 h at 4 °C, dehydrated with a graded series of EtOH (30%, 50%, 70%, 90% and 100%). Subsequently, EBs were embedded and sectioned. TEM images were taken using the JEM-1400 Flash Electron Microscope at the Indiana Center for Biological Microscopy.

### Western blotting

Cells were lysed using the Complete Lysis-M EDTA-free kit (Roche, 04719964001). Lysates were run on Mini-PROTEAN TGX Precast Gels (Bio-Rad) and transferred to polyvinylidene difluoride membranes using a Trans-Blot Turbo Transfer System (Bio-Rad). The membrane was blocked with 5% non-fat milk for 1 h at room temperature, incubated in TBXT buffer containing 5% BSA and primary antibodies against MYH10 (1:1,000, Santa Cruz, sc-33729), FLAG (1:1,000, Cell Signaling, 14793S), ATGL1 (1:1,000, Life Technologies, 55190-1-AP), PLIN5 (1:1,000, Life Technologies, 26951-1-AP) or GPAM (1:1,000, Life Technologies, PA520524) at 4 °C overnight. The membrane was then washed with TBXT and incubated with horseradish peroxidase-conjugated anti-rabbit IgG (1:200, Santa Cruz Biotechnology, sc-2357) or anti-mouse IgG (Santa Cruz Biotechnology, sc-2005) secondary antibody, and detected using ECL Western Blotting Substrate (Pierce) on a ChemiDoc Imaging System (Bio-Rad).

### Echocardiography

Mice were anaesthetized with 1–2% isoflurane, and chest hairs were shaved. The anaesthetized mice were placed on a heated platform in the prone position. Pre-heated ultrasound coupling gel was applied to the chest area, and a linear array transducer (18–23 MHz) was positioned to obtain one-dimensional M-mode images or two-dimensional B-mode parasternal long- and short-axis views on a VisualSonics Vevo 2100 Imaging System. Left ventricle FS percentages and EF percentages were calculated from the M-mode measurements using Vevo LAB ultrasound analysis software at the IU Small Animal Ultrasound Core.

### AAV9 transgene

Dr Zhiqiang Lin (Masonic Medical Research Institute, United States) provided the AAV9-CTNT vector. AAV9-CTNT-GFP (green fluorescent protein), AAV9-CTNT-AS-*LIPTER* and AAV9-CTNT-*LIPTER* (human full-length LIPTER RNA) contained a chicken troponin T promoter to drive CM-specific GFP, AS-*LIPTER* and *LIPTER* expression. AAV9 viruses were packaged by the Genome Editing, Transgenic, and Virus Core at Magee-Women’s Research Institute with a fee-based service. AAV9 was administered by retro-orbital injection into 6-week-old B6.BKS(D)-Lepr^db^/J mice (stock no. 000697, The Jackson Laboratory) at 2 × 10^10^ vg/g. WT C57BL/6J mice (stock no. 000664, The Jackson Laboratory) without AAV9 injection were also included as a control.

### *NKX2.5* knockdown

AAV9-shNKX2.5 were utilized to knock down *NKX2.5* in hiPSC-derived CMs. Briefly, AAV9-shRNA-ctrl (Addgene, 85741) was used as a control scramble shRNA. Two shRNAs targeting human *NKX2.5* were designed and inserted into the AAV9 plasmid to replace the shRNA-ctrl sequence. The pAdDeltaF6 and AAV2/9 plasmids were used for packaging viruses. Viruses with a concentration of 1 × 10^7^ GC ml^−1^ were used to infect hiPSC-derived EBs. Media containing AAV9 virus were changed every three days and maintained for 9 days.

### Cryosection and CM area measurement

HiPSC-derived EBs were fixed with 4% PFA for 30 min, washed with PBS and dehydrated in sucrose solutions of increasing concentration (10%, 20% and 30%) for 1 h each. Dehydrated EBs were embedded in OCT (Fisher Scientific, 23730571) and stored at −80 °C. Frozen EBs were sectioned at 7 μm on a Leica CM3050 cryostat and stored in a −80 °C freezer. For human heart LV tissues and mouse heart sectioning, the tissues were fixed with 4% PFA overnight, and dehydrated in sucrose solutions of increasing concentration (10%, 20% and 30%) for 3 h each. Fixed tissues were embedded in OCT and stored at −80 °C freezer. To measure myocyte cross-sectional area, the sections were stained with Alexa Fluor 488-conjugated wheat germ agglutinin (Invitrogen, W11261) for membrane and with DAPI for nuclei. A minimum of 50 CMs per section were manually outlined and single-cell cross-sectional area was measured using ImageJ software (National Institutes of Health (NIH)).

### Fast Green and Picoro-Sirius Red staining

Mouse heart sections were fixed with Bouins’ solution at 55 °C for 1 h and washed with tap water for 45 min. Until yellow colour disappeared, sections were stained with 0.1% Fast Green for 30 min, followed by 1% acetic acid for 15 s and tap water for 2 min. Sections were then stained with 0.1% Sirius Red for 30 min, dehydrated with EtOH (70–100%), cleared with xylene and mounted with Cytoseal 60 (Fisher Scientific, 23-244257).

### RNA FISH

Cryosections of hiPSC-EBs and human heart tissues were prepared by following the sample preparation protocol for fixed frozen tissues from ACD Inc. Briefly, the tissue sections were washed with PBS, baked at 60 °C for 30 min, post-fixed with 4% PFA for 15 min at 4 °C, and then dehydrated with 50%, 70% and 100% EtOH for 1 × 5 min at room temperature. Tissues were baked in target retrieval solution at 98–102 °C for target retrieval, and then treated with Protease IV for 30 min at 40 °C. The *LIPTER* specific probes labelled with C2 and negative control probes were synthesized at ACD Inc. The prepared tissue and EB sections were hybridized with *LIPTER* or control probes following the RNAscope Fluorescent Multiplex Assay protocol. Briefly, the sections were incubated with Amp 1-FL for 30 min, Amp 2-FL for 15 min, Amp 3-FL for 1 × 30 min and Amp4 AltB-FL for 15 min at 40 °C, and washed with Wash Buffer twice at room temperature between each inculcation. For FISH and lipid double stainings, after FISH, the sections were stained with 1:1,000 LipidTOX Deep Red (ThermoFisher, H34477) in PBS for 30 min at room temperature. After three washes with PBS, samples were mounted with DAPI Fluoromount-G (SouthernBiotech, 0100-20) for imaging.

### Immunofluorescent microscopy

EBs or tissue sections were washed with PBS to remove OCT and blocked with 5% goat serum in PBST for 1 h at room temperature. CMs were fixed with cold 4% PFA for 15 min at room temperature. The blocked sample sections and fixed CMs were incubated with anti-cTnT antibody (1:200, Thermo Fisher Scientific, MS-295-P), cleaved caspase-3 antibody (1:200, Cell Signaling, 9664), MYH10 antibody (1:200, Santa Cruz, sc-33729), NKX2.5 antibody (1:100, Abcam, ab97355), RXRa antibody (1:200, ABclonal Science, A19105), or CEBPB antibody (1:100, Abcam, ab32358) in blocking buffer containing 2% goat serum and 0.1% saponin overnight at 4 °C. After incubation, samples were washed with PBS three times and incubated with fluorescence-labelled secondary antibody (1:200, Thermo Fisher Scientific, A-21235, A-11001 or A-21422) for 1 h at 37 °C. Finally, cells were washed three times in 1× PBS, then mounted with DAPI Fluoromount-G (SouthernBiotech, 0100-20) for imaging.

### Lipid staining

Samples were treated with Oil Red O (Sigma, O0625) staining or Nile Red (Sigma, 19123) staining to detect LDs. For Oil Red O staining, samples were washed in 60% isopropanol for 5 min and then incubated with Oil Red O working solution (300 mg Oil Red O in 100 ml isopropanol, and 6:4 diluted with water) for 20 min at room temperature. After washing with 60% isopropanol for 1 min and H_2_O for 5 min, the slides were mounted with DAPI Fluoromount-G (SouthernBiotech, 0100-20) for imaging. For Nile Red Staining, slides were stained with Nile Red (Sigma, 19123) at 1 μg ml^−1^ for 10 min at room temperature, washed with PBS and mounted with DAPI Fluoromount-G (SouthernBiotech, 0100-20).

### TUNEL staining

TUNEL staining was performed following the manufacturer’s protocol of In Situ Cell Death Detection Kit (TMR red) (Roche, 12156792910). Briefly, after immunofluorescent staining with anti-cTnT antibody (1:200, Thermo Fisher Scientific, MS-295-P), heart tissue sections or cell samples were permeabilized with permeabilization solution (0.1% Triton X-100 in 0.1% sodium citrate) for 2 min on ice and washed with PBS twice. Samples were then stained with TUNEL reaction mixture for 1 h at 37 °C. After washing with PBS three times for 5 min each, the samples were mounted with DAPI Fluoromount-G medium (SouthernBiotech, 0100-20) for imaging.

### RNA isolation, cDNA synthesis and RT–qPCR

Total RNAs were extracted from cells or tissues using TRIzol Reagent (Invitrogen, 15596018) and purified with the RNeasy mini kit (Qiagen, 74106). DNase treatment was performed using RNase-free DNase (Qiagen) to remove any contaminating genomic DNA. Total RNAs were reverse transcribed into complementary DNA using the High-Capacity cDNA Reverse Transcription Kit (Applied Biosystems, 4374966). RT–qPCR was performed on a QuantStudio 6 Flex system (Applied Biosystems) using SYBR Green Master Mix (Applied Biosystems, 4385614). Results were analysed using the 2^−ΔΔCt^ method and normalized to the expression of *ACTB* gene. RT–PCR was performed with Thermo Fisher Scientific DreamTaq Green PCR Master Mix. Primer sequences are described in Supplementary Table 6.

### RNA–lipid overlay assay

The RNA–lipid overlay assay was performed according to the previously published method^58^. Briefly, Biotinylated *LIPTER* RNA, truncated *LIPTER* RNA fragments and control AS-*LIPTER* were transcribed using the TranscriptAid T7 High Yield Transcription Kit (ThermoFisher, K0441), and labelled with RNA 3′ End Biotinylation Kit (ThermoFisher, 20160) following the manufacturer’s protocols. Lipid strips membrane (Echelon Biosciences, P-6002) was blocked with RNA–lipid binding buffer and then incubated with biotinylated RNA (1 µg ml^−1^) in RNA–lipid binding buffer supplemented with 50 U ml^−1^ RNase inhibitor. The membrane was then washed three times for 10 min with RNA–lipid binding buffer supplemented with 0.05% IGEPAL CA-630 (Sigma, I8896), and signal was detected using the Chemiluminescent Nucleic Acid Detection Module (ThermoFisher, 89880) of a ChemiDoc Imaging System (Bio-Rad).

### Giant lipid vesicles assay

To generate giant lipid vesicles^59^, fluorescent-labelled phospholipids including TopFluor TMR PA (Avanti Polar Lipids, 810240), TopFluor PI4P (Avanti Polar Lipids, 810185) and TopFluor PI (Avanti Polar Lipids, 810187) were dissolved in CHCl_3_. A total of 250 nmol of each fluorescent-labelled PA, PI4P or PI in 500 µl CHCl_3_ was transferred to a small glass tube (ThermoFisher, 14-958) and dried using nitrogen air in a chamber. A thin lipid layer was formed on the bottom of the glass bottle. RNA–lipid binding buffer was slowly added to the bottle, and after 48 h of incubation at room temperature in the dark, giant lipid vesicles were collected from the upper layer of the solution. *LIPTER*, *LIPTER*-antisense and LIPTER exon 1–2 and exon 3 fragments were labelled with the Alexa Fluor 594 Nucleic Acid Labeling Kit (ThermoFisher, U21654) according to the manufacturer’s protocol. The lipid vesicles and the Alexa594-labelled RNAs were gently mixed at a volume ratio of 5:1, incubated at room temperature for 15 min in the dark, and dropped on glass coverslips. Images were captured using a DMi8 inverted fluorescent microscope (Leica).

### MST assay

MST assays were performed on a NanoTemper Monolith NT.115 with blue/red filters (NanoTemper Technologies) at the Physical Biochemistry Instrumentation Facility at Indiana University, Bloomington. TopFluor TMR PA (Avanti Polar Lipids, 810240) and TopFluor PI4P (Avanti Polar Lipids, 810185) were dissolved in RNA–lipid binding buffer (100 nM), and a final concentration of 25 nM of each was used. *LIPTER* and *LIPTER*-antisense RNA were synthesized in vitro and serially diluted (1–9,600 nM) in the RNA–lipid binding buffer into 16 vials with 10 µl per vial. Fluorescent-labelled PA or PI4P (10 µl) was added and mixed well in each vial. Next, 10 µl of the mixed solution from each vial was removed and loaded into a capillary. Fluorescence intensities of the 16 capillaries were measured at 40% MST power and 90% LED power. The *K*_d_ value was calculated using MO Affinity Analysis software.

### MS2-tagged RNA affinity purification

MS2-tagged RNA affinity purification was performed as previously described^60^. Plasmids pcDNA3-FLAG-MS2 and pcDNA3-24xMS2-stemloop were obtained from Dr Je-Hyun Yoon Lab (Medical University of South Carolina, United States). *LIPTER* full-length RNA, *LIPTER* RNA fragments and control RNA were cloned into the pcDNA3-24xMS2-stemloop plasmid between HindIII and EcoRI sites. HEK293T cells (CRL-3216, ATCC) were co-transfected with 6 µg pcDNA3-*LIPTER*-24xMS2SL (or different exons-24xMS2SL or pcDNA3-LIPTER-antisense-24xMS2SL) plasmids and 3 µg pcDNA3-FLAG/YFP-MS2 by using X-tremeGENE 9 DNA Transfection Reagent (Roche, 6365787001). After 48 h, transfected cells were collected to pull down RNA binding proteins using the EZ-Magna RIP RNA-Binding Protein Immunoprecipitation Kit (Sigma,17701). Cells were lysed with 100 µl RIP lysis buffer plus RNase inhibitor with protease inhibitor cocktail. Then 100 µl cell lysate was mixed with 860 µl RIP wash buffer, 5 µg FLAG antibody (1:200, Cell Signaling, 14793S)-labelled magnetic beads, 35 μl 0.5 M EDTA and 5 μl RNase inhibitor and incubated. The mixture was rotated overnight at 4 °C. After inculcation, beads were washed with RIP wash buffer six times, resuspended in RIPA lysis buffer plus loading buffer and heated for 5 min at 95 °C. The beads were removed by centrifuge at 200*g* for 5 min, and the supernatant was collected for SDS–PAGE, followed by silver staining using the Pierce Silver Stain kit (ThermoFisher, 34612) or western blotting.

### Live cell imaging

To trace *LIPTER* RNA in live CMs, *LIPTER*-24xMS2SL and *LIPTER*-antisense-24xMS2SL were separately inserted into a pHAGE lentiviral vector with puromycin resistance. The pHR-tdMCP-YFP (MS2-coat binding protein tagged with YFP) lentiviral plasmid was obtained d from Addgene (plasmid number 99151). A total of 1 × 10^6^ hiPSCs were infected with packaged pHAGE-LIPTER-24xMS2SL or pHAGE-LIPTER-antisense-24xMS2SL lentivirus for 72 h. After infection, cells were selected using 1 µg ml^−1^ puromycin for 4 days. The remaining cells were then infected with pHR-tdMCP-YFP lentivirus. YFP^+^ cells were sorted by a BD FACSAria II Cell Sorter, expanded and differentiated into CMs. The differentiated CMs were seeded into poly-d-lysine-coated 35 mm glass-bottom dishes (MatTek, P35GC-0-10-C). Live cell video was taken on a Nikon live cell imaging system with an Apo 60× Oil lens at Indiana Center for Biological Microscopy. Consecutive images were taken during 4 h with 3 min intervals, and a total of 81 images were taken per field. The video was exported with 16 frames per second using the NIS Elements software.

### Quantifications of FA and TAG in mouse hearts

The concentration of free FA was quantified by using the Free Fatty Acid Assay kit (Abcam, ab65341), and TAG concentrations were quantified using a Triglyceride Quantification Assay Kit (Abcam, ab65336). Approximately 25 mg of mouse heart tissue per heart was collected and homogenized in 500 μl lipid extraction buffer (Abcam, ab211044) using a Beadbug Homogenizer. After centrifugation, the supernatant was removed to a new tube. FA or TAG concentration was measured according to the manufacturer’s protocols. The FA and TAG concentrations in WT and genetically modified mouse hearts were normalized to the collected heart tissue weights.

### Whole mouse heart FAO rate assay

Freshly collected mouse heart was minced into small pieces and thoroughly homogenized in ice-cold STE buffer (0.25 M sucrose, 10 mM Tris–HCl and 1 mM EDTA, at pH of 7.4) using a Dounce homogenizer. After homogenization, the specimen was centrifuged at 420*g* at 4 °C for 10 min. Supernatant containing crude mitochondria was transferred into chilled Eppendorf tubes for protein quantification and FAO assay as previously described^61–63^. In brief, 30 µl of tissue homogenate was added into Eppendorf tubes in triplicate (including STE blank control for background reading), followed by the immediate addition of 370 µl oxidation reaction mixture (100 mM sucrose, 10 mM Tris–HCl, 5 mM KH_2_PO_4_, 0.2 mM EDTA, 80 mM KCl, 1 mM MgCl_2_, 2 mM l-carnitine, 0.1 mM malate, 0.05 mM Coenzyme A, 2 mM ATP, 1 mM dithiothreitol, 0.5 mM palmitate, 0.7% BSA and 0.4μCi ^14^C-palmitate), and incubated at 37 °C for 1 h. After incubation, the entire reaction mixture was transferred into tubes containing 200 µl of 1 M perchloric acid and the CO_2_ trap disc, and incubated for 1 h at room temperature. Then the trap disc was transferred to scintillation vials for complete oxidation measurement, whereas the supernatant obtained by centrifugation of the remaining acid solution was used to measure acid-soluble metabolites (ASMs) produced by incomplete oxidation. In addition, the nonincubated reaction mixture was counted in triplicate to get the measurement of the amount of radioactivity input into each reaction.

The specific activity of the reaction mixture was determined by dividing the disintegrations per minute (DPM) of the input by the number of nanomoles of palmitate (cold + hot) per reaction. The specific activity can be used to convert DPM to nanomoles for the other samples.

$${\mathrm{Specific}}\,{\mathrm{activity}}=\frac{{\mathrm{DPM}}\,{\mathrm{of}}\,{\mathrm{input}}}{{\mathrm{Total}}\,{\mathrm{nmol}}\,{\mathrm{of}}\,{\mathrm{palmitate}}}$$

To determine the rate of conversion of ^14^C-palmitate to ^14^CO_2_, we divide the nanomoles of ^14^CO_2_ by the amount of time in the reaction and the amount of protein per action. A similar calculation can be done to determine the rate of conversion of ^14^C-palmitate to ASMs.

$$\begin{array}{l}{\mathrm{FAO}}\,{\mathrm{rate}}=\frac{({\mathrm{DPM}}\,{\mathrm{of}}\,{\mathrm{sample}})-({\mathrm{DPM}}\,{\mathrm{of}}\,{\mathrm{background}})}{({\mathrm{Specific}}\,{\mathrm{activity}})\times ({\mathrm{reaction}}\,{\mathrm{time}})\times ({\mathrm{protein}}\,{\mathrm{amount}}\,{\mathrm{per}}\,{\mathrm{action}})}\end{array}$$

### Untargeted metabolomics

Untargeted metabolomics was performed, and data were analysed by Creative Proteomics. Briefly, 10 million enriched hiPSC-CMs were collected at day 40 and washed with PBS. Metabolites were extracted with 800 μl of methanol, 10 μl of DL-o-chlorophenylalanine (2.8 mg ml^−1^) and 10 μl of LPC (12:0), followed by ultrasonication for 30 min. The samples were analysed using the Ultimate 3000 HPLC and UHPLC combined with Q Exactive MS systems (ThermoFisher) and screened with electrospray ionization (ESI)–MS. Raw data were acquired and aligned using the Compound Discover (3.0, ThermoFisher) based on the *m*/*z* value and the retention time of the ion signals. Ions from both ESI^−^ and ESI^+^ were merged and imported into the SIMCA-P program (version 14.1) for multivariate analysis.

### Seahorse mitochondrial function assays

Seahorse XFp Extracellular Flux Analyzer was used to measure oxygen consumption rate (OCR) and extracellular acidification rate of hiPSC-CMs according to the manufacturer’s protocol. Briefly, 5 × 10^4^ CMs (based on FCCP titration assay) were seeded into Matrigel-coated eight-strip utility plate for 48 h before Mito Stress Test assay. We prepared the pH 7.4 assay medium (with 4 mM l-glutamine and 25 mM glucose),and hydrated the cartridge in calibration medium at 37 °C without CO_2_ before running the assay. Then 1.5 μM oligomycin was loaded into port A, 0.25 μM FCCP into port B and 0.5 μM Rot/AA into port C on the cartridge. The Mito Stress Test was run according to the standard process of analyzer. After the assay, the protein concentration of each well was measured using BCA protein assay kit (ThermoFisher, 23225). Raw data were processed using Seahorse Wave software.

Long-chain FAO was measured according to the protocol of Palmitate Oxidation Stress Test Kit on a Seahorse XF96 Analyzer. Briefly, 5 × 10^4^ CMs were seeded into one well of Matrigel-coated 96-well utility microplate, cultured in normal culture medium for 48 h and then switched to the substrate-limited growth medium (DMEM plus 0.5 mM glucose, 1.0 mM GlutaMAX, 1% FBS and 0.5 mM l-carnitine) overnight. The FAO assay buffer was prepared according to the protocol and replaced the substrate-limited growth medium 45 min before measurement. FAO buffer or 40 μM etomoxir was added into each well 15 min before measurement. BSA control or palmitate:BSA was added into well before the Mito Stress test was started. FAO level was quantified on the basis of the differences in the values of the maximal oxygen consumption in palmitate:BSA without or with presence of etomoxir.

### Illumina RNA sequencing and data analysis

Transcriptional profiles of human ESC-derived CMs, SMCs and ECs were obtained by Illumina mRNA deep sequencing using a service from LC Sciences as we previously reported^24^. Three non-failure hearts, three non-failure hearts with T2DM, three hearts with DCM, and three hearts with DCM and T2DM were subject to mRNA-seq. Briefly, total RNAs were extracted from human left ventricle tissues by using the RNeasy kit (Qiagen, 74106), and sequenced by using paired-end deep-transcriptome sequencing with the Illumina platform at IU Genomic Core. All lncRNAs were profiled according to their fragments per kilobase of transcript per million mapped reads values. Additionally, mRNAs were extracted from enriched day 40 WT and *LIPTER*^KO^ hiPSC-CMs, as well as hearts of WT and *LIPTER*(Tg) mice fed with HFD for 7 months and sequenced by using paired-end deep-transcriptome sequencing with the Illumina platform. The RNA-seq data were collected and analysed as we previously described^24,64^. In general, the sequencing reads were mapped to the reference genome (either hg38 or mm10) by STAR (version 2.7.2a). Gene expression levels were evaluated by the featureCounts on uniquely mapped reads. Following gene expression normalization based on trimmed mean of M (TMM) values, edgeR (refs. ^65,66^) was employed to perform differential analysis given the comparison between *LIPTER*^KO^ and WT for human samples or *LIPTER*(Tg) and WT for mouse heart samples. The genes with false discovery rate (FDR)-adjusted *P* values <0.05 after multiple test correction were determined as differentially expressed genes.

### Functional enrichment analysis

GO analysis was conducted on differentially expressed genes using DAVID^67–69^. The gene set enrichment analysis (GSEA)^70^ was performed (http://www.gsea-msigdb.org/gsea/index.jsp, V4.1.0) based on the fold change (log_2_) of gene expression between either KO versus WT CMs or Tg versus WT hearts by focusing on C5 biological process (bp) from GO database or specific toxicity gene sets retrieved from Ingenuity Pathway Analysis^71^. The significantly enriched GO terms or toxicity-related gene sets were selected for presentation in figures.

### RIP

RIP was conducted using the EZ-Magna RIP RNA-Binding Protein Immunoprecipitation kit (Millipore) according to the manufacturer’s instruction (Sigma,17701). Briefly, 1 × 10^6^ hiPSC-CMs were resuspended in 50 μl of RIP lysis buffer with protease inhibitor cocktail and RNase inhibitor. For each sample, 5 μg anti-human MYH10 antibody (1:400, Abcam, ab230823) was added and 1 μg isotype IgG antibody (1:200, Cell Signaling, 5415S) was used as the control. RIP–qPCR results were calculated as fold enrichment from specific antibody versus non-specific IgG (1:200, Cell Signaling, 5415S).

### RIP-seq for mouse hearts

RNA immunoprecipitation was performed with mouse hearts as described above. Hearts were collected from WT and LIPTER (Tg) mice. One-hundred milligrams of left ventricle tissue per heart was collected and lysed with RIP lysis buffer according to the manufacturer’s protocol (Sigma, 17701). Anti-Myh10 antibody (1:400, Abcam, ab230823) was used to pull down Myh10-binding RNAs. Normal rabbit IgG was used as negative control. The pulldown RNAs were extracted by using the RNeasy mini kit (Qiagen, 74106). Total RNA sequencing was performed at the IU Genomic Core. The sequence reads were mapped to the mouse genome mm10 using STAR (v2.7.2a) with the the following parameter: ‘–outSAMmapqUnique 60’. Then unmapped reads were mapped to the human genome hg38 by STAR with the same parameter. Peak calling on mapped reads was performed by MACS2 compared with IgG signals with a cut-off of FDR-adjusted *P* value <0.01.

### ScRNA-seq data analysis

The gene count matrix of scRNA-seq data from a foetal human heart was retrieved from a previously published report^25^. Normalization, dimensionality reduction and clustering of scRNA-seq data were performed using Seurat (v3) (ref. ^72^) according to the parameters previously utilized^25^. The *t*-distributed stochastic neighbour embedding was used to visualize single-cell clusters, and gene expression in a reduced 2D space. Annotation of cell clusters was mapped from the annotation of the clusters presented in this report^25^.

### Statistics and reproducibility

Statistical results were analysed using Prism 8 (GraphPad Software). Unpaired Student’s *t*-tests (two groups) and two-way analysis of variance were used in data analyses unless specified in the legend. Column bar plots show mean ± standard error of the mean (s.e.m.) from at least three independent experiments. Statistical parameters, including the exact value of *n*, statistical test method and statistical significance, are reported in the figures, figure legends and related data resources. Data were judged to be significant when *P* < 0.05. No statistical method was used to pre-determine sample size. No animal or data were excluded from the analyses. The experiments were not randomized. The Investigators were not blinded to allocation during experiments and outcome assessment.

### Reporting summary

Further information on research design is available in the Nature Portfolio Reporting Summary linked to this article.

## Online content

Any methods, additional references, Nature Portfolio reporting summaries, source data, extended data, supplementary information, acknowledgements, peer review information; details of author contributions and competing interests; and statements of data and code availability are available at 10.1038/s41556-023-01162-4.

## Supplementary information

Supplementary Information Flow cytometry gating for cTnT positive. hiPSC-EBs were dissociated into single cells and then analysed by flow cytometry.

Reporting Summary

Supplementary Tables Supplementary Tables 1–6.

Supplementary Video 1 Stacked images from confocal microscopy showing co-localization of *LIPTER*, MYH10 and LDs in hiPSC-CMs. 3D video indicates *LIPTER*-MS2^YFP^ (green), MYH10 (red), LDs (magenta) and DAPI (blue) staining in WT hiPSC-CMs.

Supplementary Video 2 *LIPTER* and LDs migrating together in hiPSC-CMs. Video showing *LIPTER*-MS2^YFP^ (green) and Rhodamine-B-labeled-LDs (red) migrating together (yellow dots) only in cytosol of WT hiPSC-CMs. N, nucleus.

## Data Availability

RNA-seq data of WT and *LIPTER*^KO^ hiPSC-CMs, and WT and *LIPTER* (Tg) mouse hearts were deposited in NCBI GEO database (GSE175370). The UCSC Genome Browser view of RNAseq results from T2DM and NF human hearts can be found at https://genome.ucsc.Edu/s/samal/hg38_YangLei_ILMN550. The metabolomics data can be accessed in Supplementary Table 1. The datasets used to determine *LINC00881* expression in human hearts can be accessed at NCBI Accession GSE64283 as shown in Extended Data Fig. 1c^73^, NCBI Accession PRJNA280600 as shown in Extended Data Fig. 1d^74^ and NCBI Accession GSE30611 (Illumina Human Body Map 2.0 Project) as shown in Extended Data Fig. 1e. Source data are provided with this paper. All other data supporting the findings of this study are available from the corresponding author on reasonable request.

## Source data

Source Data Fig. 1 Statistical source data.
Source Data Fig. 2 Statistical source data.
Source Data Fig. 3 Statistical source data.
Source Data Fig. 4 Statistical source data.
Source Data Fig. 5 Statistical source data.
Source Data Fig. 6 Statistical source data.
Source Data Fig. 7 Statistical source data.
Source Data Extended Data Fig. 1 Statistical source data.
Source Data Extended Data Fig. 2 Statistical source data.
Source Data Extended Data Fig. 3 Statistical source data.
Source Data Extended Data Fig. 4 Statistical source data.
Source Data Extended Data Fig. 5 Statistical source data.
Source Data Extended Data Fig. 6 Statistical source data.
Source Data Extended Data Fig. 7 Statistical source data.
Source Data Extended Data Fig. 8 Statistical source data.
Source Data Extended Data Fig. 9 Statistical source data.
Source Data Extended Data Fig. 10 Statistical source data.
Source Data Extended Data Fig. 5 Unprocessed western blots and/or gels.
Source Data Extended Data Fig. 2 Unprocessed western blots and/or gels.
Source Data Extended Data Fig. 4 Unprocessed western blots and/or gels.
Source Data Extended Data Fig. 7 Unprocessed western blots and/or gels.
