# Supplementary material for: Lipid droplet-associated lncRNA LIPTER preserves cardiac lipid metabolism
Source: Nat Cell Biol. 2023 Jun 1;25(7):1033–46. doi: 10.1038/s41556-023-01162-4 (PMC10344779; doi:10.1038/s41556-023-01162-4)
Supplement: Supplementary file 1 — Flow cytometry gating for cTnT positive. hiPSC-EBs were dissociated into single cells and then analysed by flow cytometry. [file 41556_2023_1162_MOESM1_ESM.pdf]

nature cell biology

Article

https://doi.org/10.1038/s41556-023-01162-4

Lipid droplet-associated lncRNA *LIPTER* preserves cardiac lipid metabolism

In the format provided by the authors and unedited

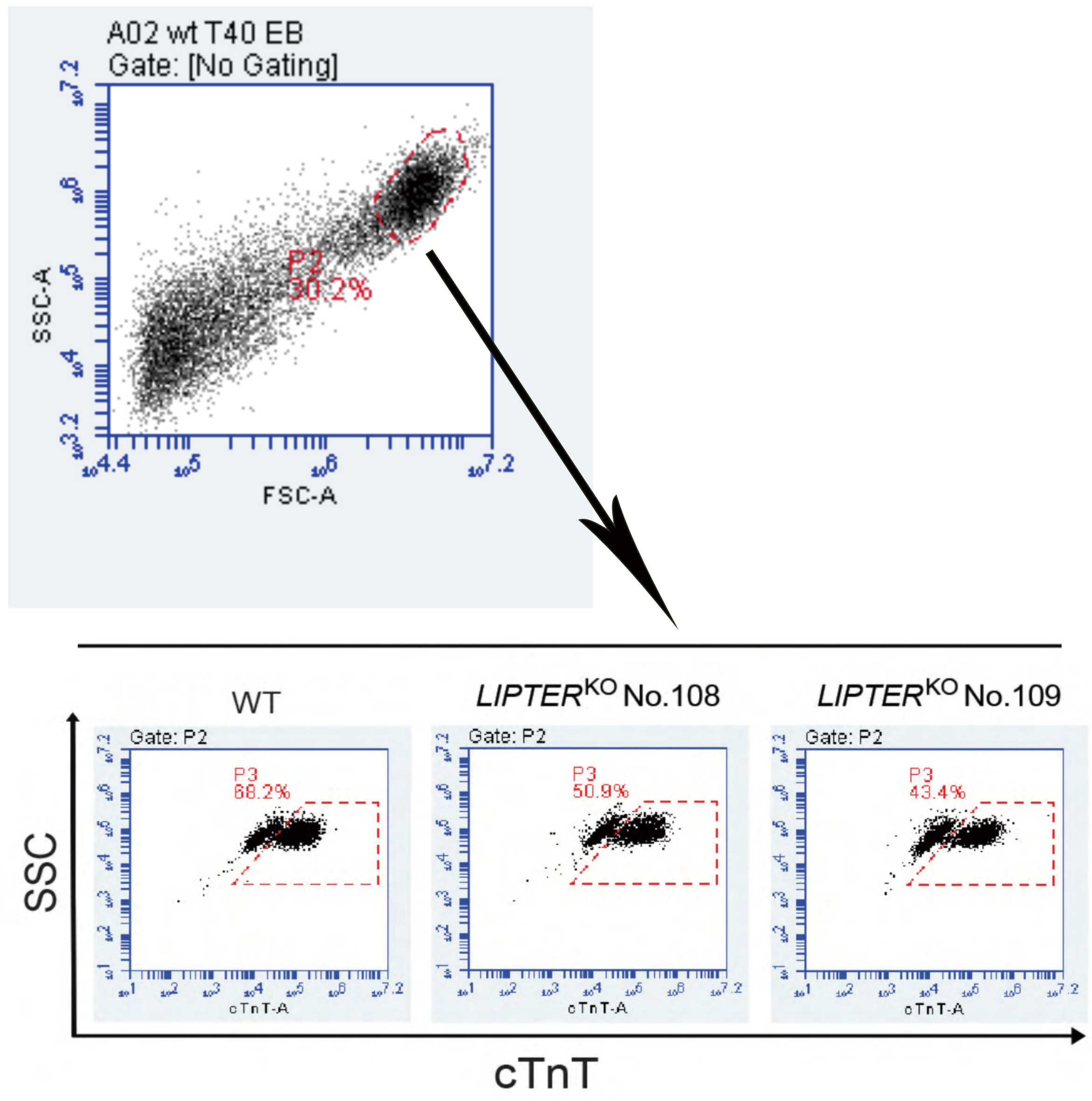

Supplementary Figure 1. Flow cytometry gating for cTnT positive. HiPSC-EBs were dissociated into single cells and then analyzed by flow cytometry.
