# Supplementary material for: Lipid droplet-associated lncRNA LIPTER preserves cardiac lipid metabolism
Source: Nat Cell Biol. 2023 Jun 1;25(7):1033–46. doi: 10.1038/s41556-023-01162-4 (PMC10344779; doi:10.1038/s41556-023-01162-4)
Supplement: Supplementary file 2 — Reporting Summary [file 41556_2023_1162_MOESM2_ESM.pdf]

nature portfolio

Corresponding author(s): Lei Yang

Last updated by author(s): Apr 20, 2023

Reporting Summary

Nature Portfolio wishes to improve the reproducibility of the work that we publish. This form provides structure for consistency and transparency in reporting. For further information on Nature Portfolio policies, see our Editorial Policies and the Editorial Policy Checklist.

Statistics

For all statistical analyses, confirm that the following items are present in the figure legend, table legend, main text, or Methods section.

n/a	Confirmed	
☐	☒	The exact sample size (n) for each experimental group/condition, given as a discrete number and unit of measurement
☐	☒	A statement on whether measurements were taken from distinct samples or whether the same sample was measured repeatedly
☐	☒	The statistical test(s) used AND whether they are one- or two-sided<br>*Only common tests should be described solely by name; describe more complex techniques in the Methods section.*
☒	☐	A description of all covariates tested
☒	☐	A description of any assumptions or corrections, such as tests of normality and adjustment for multiple comparisons
☐	☒	A full description of the statistical parameters including central tendency (e.g. means) or other basic estimates (e.g. regression coefficient) AND variation (e.g. standard deviation) or associated estimates of uncertainty (e.g. confidence intervals)
☒	☐	For null hypothesis testing, the test statistic (e.g. F, t, r) with confidence intervals, effect sizes, degrees of freedom and P value noted<br>*Give P values as exact values whenever suitable.*
☒	☐	For Bayesian analysis, information on the choice of priors and Markov chain Monte Carlo settings
☒	☐	For hierarchical and complex designs, identification of the appropriate level for tests and full reporting of outcomes
☒	☐	Estimates of effect sizes (e.g. Cohen's d, Pearson's r), indicating how they were calculated

Our web collection on statistics for biologists contains articles on many of the points above.

Software and code

Policy information about availability of computer code

Data collection	The RNA-seq and RIN-seq were performed on the lllumina HiSeq 2500. Mouse heart functions were collected by using a Vevo 2100 high-resolution digital imaging platform. Images of histological results were collected on a Leica DMi8 inverted fluorescent microscope built-in Leica Application Suite (LASX) software. Sea horse data analysis was done using Agilent proprietary software. Live cell video was taken on a Nikon live cell imaging system with built-in Nikon Application. RT-qPCR was performed on a QuantStudio 6 Flex system (Applied Biosystems).
Data analysis	RNA-seq: In general, the sequencing reads were mapped to the reference genome (either hg38 or mm10) by STAR [Version 2.7.2a]. Gene expression levels were evaluated by the featureCounts on uniquely mapped reads. Following gene expression normalization based on trimmed mean of M (TMM) values, the edgeR was employed to perform differential analysis given the comparison between LIPTER KO and WT for human samples or LIPTER(Tg) and WT for mouse samples.<br>Untargeted metabolomics: Data were acquired and aligned by using the Compound Discover (3.0, ThermoFisher) based on the m/z value and the retention time of the ion signals. Ions from both ESI- and ESI+ were merged and imported into the SIMCA-P program (version 14.1) for multivariate analysis.<br>ScRNA-seq: Normalization, dimensionality reduction, and clustering of scRNA-seq data were performed using the Seurat (v3). The t-distributed Stochastic Neighbor Embedding (tSNE) were used to visualize single-cell clusters, and gene expressions in a reduced 2D space.<br>Flow cytometry: Data were analysed using FlowJO.<br>GO analyses were performed using GSEA.<br>qPCR: Ct values were calculated using QuantStudio 6. Statistical analyses were performed using Prism 8.0.

For manuscripts utilizing custom algorithms or software that are central to the research but not yet described in published literature, software must be made available to editors and reviewers. We strongly encourage code deposition in a community repository (e.g. GitHub). See the Nature Portfolio guidelines for submitting code & software for further information.

Data

Policy information about availability of data

All manuscripts must include a data availability statement. This statement should provide the following information, where applicable:

- Accession codes, unique identifiers, or web links for publicly available datasets
- A description of any restrictions on data availability
- For clinical datasets or third party data, please ensure that the statement adheres to our policy

RNA-seq data of WT and LIPTER KO hiPSC-CMs, and WT and LIPTER (Tg) mouse hearts were deposited in to NCBI with GEO Submission number GSE175370 (https://www.ncbi.nlm.nih.gov/geo/query/acc.cgi?acc=GSE175370). The UCSC Genome Browser view of RNAseq results from T2DM and NF human hearts could be found here: https://genome.ucsc.Edu/s/samal/hg38_YangLei_ILMN550.

Human research participants

Policy information about studies involving human research participants and Sex and Gender in Research.

Reporting on sex and gender	Human left ventricle tissue used in this study were obtained from the Duke Human Heart Repository (DHHR), which is a DUHS IRB approved tissue repository. Samples were procured by the DHHR in accordance with an approved DUHS IRB protocol using written informed consent. All patient information has been de-identified. Therefore, the sex and gender information were not collected. This study does not focus on sex and gender differences either.
Population characteristics	We requested heart left ventricle tissues from DHHR, which were collected from individualizes diagnosed with DCM together with or without T2DM, as well as from individuals diagnosed without heart failure and with or without T2DM.
Recruitment	We did not recruit any patients. Study materials and de-identified medical record information of the patients were obtained from the DHHR bio-repository.
Ethics oversight	Duke Human Heart Repository (DHHR) is a DUHS IRB approved tissue repository. Given that the specimens were not collected specifically for this study and the subject identifiers linked to these specimens were not requested for this study, this study is not considered a human subject research. Informed consent was obtained by DHHR. The study is compliant with all ethical regulations.

Note that full information on the approval of the study protocol must also be provided in the manuscript.

Field-specific reporting

Please select the one below that is the best fit for your research. If you are not sure, read the appropriate sections before making your selection.

☒ Life sciences ☐ Behavioural & social sciences ☐ Ecological, evolutionary & environmental sciences

For a reference copy of the document with all sections, see nature.com/documents/nr-reporting-summary-flat.pdf

Life sciences study design

All studies must disclose on these points even when the disclosure is negative.

Sample size	For animal experiments, sample size calculation was performed using EDA (experimental design assistant) ensuring a power of 0.9, a significant difference of 0.05, and a variation and effect size for each model according to personal experience.<br>No sample size calculation was performed for other experiments. The sample size was chosen according to previous publications (ref, 19,23) and current standard.
Data exclusions	No experimental data were excluded.
Replication	All experiments in main Figures have been reported as individual dots corresponding to single replicates.
Randomization	Mice treatments were decided based on genotype. Group allocation within a genotype was randomized. For all other experiments, allocation in each group was random.
Blinding	Investigators were not blinded in data collection analyses of outcomes. Blinding was not possible because cell cultures of WT, control, siRNA-treated or KO cells had to be defined, the same as WT, KO, Tg mice.

Reporting for specific materials, systems and methods

We require information from authors about some types of materials, experimental systems and methods used in many studies. Here, indicate whether each material, system or method listed is relevant to your study. If you are not sure if a list item applies to your research, read the appropriate section before selecting a response.

Materials & experimental systems

n/a	Involved in the study
☐	☒ Antibodies
☐	☒ Eukaryotic cell lines
☒	☐ Palaeontology and archaeology
☐	☒ Animals and other organisms
☒	☐ Clinical data
☒	☐ Dual use research of concern

Methods

n/a	Involved in the study
☒	☐ ChIP-seq
☐	☒ Flow cytometry
☒	☐ MRI-based neuroimaging

Antibodies

Antibodies used

Mouse monoclonal anti-MYH10 (3H2), Santa Cruz Biotechnology Cat# sc-33729, RRID:AB_628464.
Rabbit monoclonal anti-DYKDDDDK Tag (D6W5B) Rabbit mAb (Binds to same epitope as Sigma's Anti-FLAG® M2 Antibody), Cell Signaling Technology Cat# 14793, RRID:AB_2572291
Mouse monoclonal anti-GFP antibody, Abcam Cat# ab290, RRID:AB_303395.
Rabbit monoclonal anti-CEBP Beta antibody [E299], Abcam Cat# ab32358, RRID:AB_726796.
Rabbit polyclonal anti-Nkx2.5 antibody, Abcam Cat# ab97355, RRID:AB_10680260.
Rabbit monoclonal anti- Retinoid X Receptor alpha, ABclonal Cat# A19105, RRID:AB_2862598.
Rabbit monoclonal anti-Cleaved Caspase-3 (Asp175) (5A1E), Cell Signaling Technology Cat# 9664, RRID:AB_2070042.
Mouse monoclonal anti-Troponin T, Cardiac Isoform, Thermo Fisher Cat# MS-295-P, RRID:AB_61806.
Rabbit monoclonal anti-Cardiac Troponin T [EPR20266], Abcam Cat# Ab209813.
Anti-Wheat Germ Agglutinin Conjugates Alexa Fluor 488, Invitrogen Cat# W11261.
Rabbit polyclonal anti-ATGL, Thermo Fisher Cat# 55190-1-AP, RRID:AB_11182818.
Rabbit polyclonal anti-Perilipin 5, Thermo Fisher Cat# 26951-1-AP, RRID:AB_2880699.
Rabbit polyclonal anti-GPAM, Thermo Fisher Scientific Cat# PA5-20524, RRID:AB_11155813.
Goat anti-Mouse IgG (H+L) Cross-Adsorbed Secondary Antibody, Alexa Fluor 488, Thermo Fisher Scientific Cat# A-11001, RRID:AB_2534069.
Goat anti-Mouse IgG (H+L) Cross-Adsorbed Secondary Antibody, Alexa Fluor 555, Invitrogen Cat# A-21422, RRID:AB_141822.
Goat anti-Mouse IgG (H+L) Cross-Adsorbed Secondary Antibody, Alexa Fluor 647, Thermo Fisher Scientific Cat# A-21235, RRID:AB_2535804.
Goat anti-Rabbit IgG (H+L) Cross-Adsorbed Secondary Antibody, Alexa Fluor 555, Invitrogen, Cat# A-21428, RRID:AB_141784.
Goat Anti-Rabbit IgG H&L Alexa Fluor 488, Abcam Cat# ab150077, RRID:AB_2630356.
Mouse anti-rabbit IgG-HRP, Santa Cruz Biotechnology Cat# sc-2357, RRID:AB_628497.
Goat anti-mouse IgG-HRP, Santa Cruz Biotechnology Cat# sc-2005, RRID:AB_631736.
Mouse isotype IgG control antibody, Cell Signalling Cat#5415S, RRID:AB_10829607.

Dilution:
For western blot: Antibody was diluted 1:1000.
For IF staining: Antibody was diluted range 1:50 to 1:200.
Fluorescent secondary antibody was diluted 1:200.
HRP-conjugated antibody was diluted 1:5000.

Validation

The validations were provided by the manufacturer's website.
Mouse monoclonal anti-MYH9/10 (3H2): https://datasheets.scbt.com/sc-33729.pdf
Rabbit monoclonal anti-DYKDDDDK Tag (D6W5B) Rabbit mAb :
https://www.cellsignal.com/products/primary-antibodies/dykddddk-tag-d6w5b-rabbit-mab-binds-to-same-epitope-as-sigma-s-anti-flag-m2-antibody/14793?gclid=EAIaIQobChMIufmayv3A_gIVCu3jBx3S8wIIEAAYASAAEgJP1_D_BwE&gclsrc=aw.ds.
Mouse monoclonal anti-GFP antibody, https://www.abcam.com/products/primary-antibodies/gfp-antibody-ab290.html
Rabbit monoclonal anti-CEBP Beta antibody [E299]:
https://www.abcam.com/products/primary-antibodies/cebp-beta-antibody-e299-c-terminal-ab32358.html.
Rabbit polyclonal anti-Nkx2.5 antibody: https://www.abcam.com/products/primary-antibodies/nkx25-antibody-ab97355.html.
Rabbit monoclonal anti- RXRα, https://abclonal.com/catalog-antibodies/RXRRabbitmAb/A19105
Rabbit monoclonal anti-Cleaved Caspase-3 (Asp175) (5A1E): https://www.cellsignal.com/products/primary-antibodies/cleaved-caspase-3-asp175-5a1e-rabbit-mab/9664.
Mouse monoclonal anti-Troponin T: https://www.fishersci.com/shop/products/lab-vision-troponin-t-cardiac-isoform-ab-1-mouse-monoclonal-antibody-200-g-ml-bsa-azide/p-4563797.
Rabbit monoclonal anti-Cardiac Troponin T: https://www.abcam.com/products/primary-antibodies/cardiac-troponin-t-antibody-epr20266-ab209813.html
Anti-Wheat Germ Agglutinin Conjugates Alexa Fluor 488: https://www.thermofisher.com/order/catalog/product/W11261?ef_id=EAIaIQobChMI4_OH34HB_gIV_efjBx1x9g4pEAAYASAAEgJBd_D_BwE:G:s&s_kwcid=AL!3652!3!447292198736!!!g!!!10506731179!
109642167491&cid=bid_pca_iva_r01_co_cp1359_pjt0000_bid00000_0se_gaw_dy_pur_con&gclid=EAIaIQobChMI4_OH34HB_gIV_efjBx1x9g4pEAAYASAAEgJBd_D_BwE.
Rabbit polyclonal anti-ATGL: https://www.thermofisher.com/antibody/product/ATGL-Antibody-Polyclonal/55190-1-AP
Rabbit polyclonal anti-Perilipin 5: https://www.thermofisher.com/antibody/product/Perilipin-5-Antibody-Polyclonal/26951-1-AP.
Rabbit polyclonal anti-GPAM: https://www.thermofisher.com/antibody/product/GPAM-Antibody-Polyclonal/PA5-20524.

Goat anti-Mouse IgG (H+L) Cross-Adsorbed Secondary Antibody, Alexa Fluor 488: https://www.thermofisher.com/antibody/product/Goat-anti-Mouse-IgG-H-L-Cross-Adsorbed-Secondary-Antibody-Polyclonal/A-11001.
Goat anti-Mouse IgG (H+L) Cross-Adsorbed Secondary Antibody, Alexa Fluor 555: https://www.thermofisher.com/antibody/product/Goat-anti-Mouse-IgG-H-L-Cross-Adsorbed-Secondary-Antibody-Polyclonal/A-21422.
Goat anti-Mouse IgG (H+L) Cross-Adsorbed Secondary Antibody, Alexa Fluor 647: https://www.thermofisher.com/antibody/product/Goat-anti-Mouse-IgG-H-L-Cross-Adsorbed-Secondary-Antibody-Polyclonal/A-21235.
Goat anti-Rabbit IgG (H+L) Cross-Adsorbed Secondary Antibody, Alexa Fluor 555: https://www.thermofisher.com/antibody/product/Goat-anti-Rabbit-IgG-H-L-Cross-Adsorbed-Secondary-Antibody-Polyclonal/A-21428.
Goat Anti-Rabbit IgG H&L Alexa Fluor 488: https://www.abcam.com/products/secondary-antibodies/goat-rabbit-igg-hl-alexa-fluor-488-ab150077.html.
Mouse anti-rabbit IgG-HRP: https://www.scbt.com/p/mouse-anti-rabbit-igg-hrp.
Goat anti-mouse IgG-HRP, https://www.scbt.com/p/goat-anti-mouse-igg-hrp.
Mouse isotype IgG control antibody, https://www.cellsignal.com/products/primary-antibodies/mouse-g3a1-mab-igg1-isotype-control/5415

Eukaryotic cell lines

Policy information about cell lines and Sex and Gender in Research

Cell line source(s): Wild type human iPSCs induced pluripotent stem cells (hiPSCs) were generated from fibroblasts of a healthy donor (Nature 465, 808-812, 2010.) and obtained via a MTA. HEK293T cells (CRL-3216, ATCC).

Authentication: PCR and Sanger sequencing were conducted to ensure there was no mutation on the PTPN11 gene (Nature 465, 808-812) of hiPSCs.

Mycoplasma contamination: All cellls were tested mycoplasma negative.

Commonly misidentified lines (See ICLAC register): Not included.

Animals and other research organisms

Policy information about studies involving animals; ARRIVE guidelines recommended for reporting animal research, and Sex and Gender in Research

Laboratory animals: LIPTER transgenic C57BL/6J mice were generated by using CRISPR/Cas9. Six-week-old male homozygous C57BL/6J Leprdb/db mice were purchased from the Jackson Laboratory (Strain #:000697). Myh10 flox(f) C57BL/6J mice were obtained from the Dr. Robert S. Adelstein lab at Laboratory of Molecular Cardiology, NHLBI4. C57BL/6J Tnnt2Cre mice were obtained from Dr. Chen Ieng Cai lab at Indiana University. Conditional Myh10 knockout in mouse cardiomyocytes were generated by cross Myh10 f/f mice with Tnnt2-MerCreMer mice. Six-week male Myh10 f/f and Myh10f/f/Tnnt2-MerCreMer mice were injected with tamoxifen (0.1 mg/gram body weight) at days 1, 3, 5 by intraperitoneal (IP) injection and then fed with high fat diet (45 kcal% fat, Research Diets Inc., D12451i) for 3 months. Six-week male LIPTER transgenic mice and age matched WT C57BL/6J mice were fed with high fat diet (45 kcal% fat, Research Diets Inc., D12451i) or normal chew (18 protein, Inotiv, 2018sx) for 7-10 months.

Wild animals: Not included.

Reporting on sex: Since male and female mice have differences in metabolism, all in vivo lipid metabolic studies only utilized male mice in this study.

Field-collected samples: Not included.

Ethics oversight: All experimental procedures involving animals in this study were reviewed and approved by the Indiana University's Institutional Animal Care and Use Committee.

Note that full information on the approval of the study protocol must also be provided in the manuscript.

Flow Cytometry

Plots

Confirm that:

- [x] The axis labels state the marker and fluorochrome used (e.g. CD4-FITC).
- [x] The axis scales are clearly visible. Include numbers along axes only for bottom left plot of group (a 'group' is an analysis of identical markers).
- [x] All plots are contour plots with outliers or pseudocolor plots.
- [x] A numerical value for number of cells or percentage (with statistics) is provided.

Methodology

Sample preparation: Briefly, day 20 or 40 EBs were harvested and dissociated with 1mg/ml Collagenase B for 30 min, followed with (0.25% trypsin-

Sample preparation	EDTA) for 5 min at 37°C. The dissociated single cells were fixed in 4% PFA for 15 min at RT and washed 3 times with PBS. Cells were incubated in blocking buffer containing 1xPBS, 10% goat serum and 0.1% saponin. Then cells were incubated with primary antibody diluted in 1xPBS with 2% BSA and 0.1% saponin for 1 hr at 37°C, following with APC labelled goat anti-mouse secondary antibody for 1 hr at 37°C.
Instrument	Attune NxT flow cytometer (Invitrogen)
Software	FlowJo (Treestar)
Cell population abundance	cTnT positive cell population abundance is normally above 10% for analysis.
Gating strategy	A population of single cells were gated based on cell size (forward scatter, FSC, versus side scatter, SSC). Abcam ab209813 antibody was used to stain cTNT positive cardiomyocytes. Supplementary Figure 1 shows the gate setting.

☒ Tick this box to confirm that a figure exemplifying the gating strategy is provided in the Supplementary Information.
