# Supplementary material for: Lipid droplet-associated lncRNA LIPTER preserves cardiac lipid metabolism
Source: Nat Cell Biol. 2023 Jun 1;25(7):1033–46. doi: 10.1038/s41556-023-01162-4 (PMC10344779; doi:10.1038/s41556-023-01162-4)

Raw image of Figure 5j

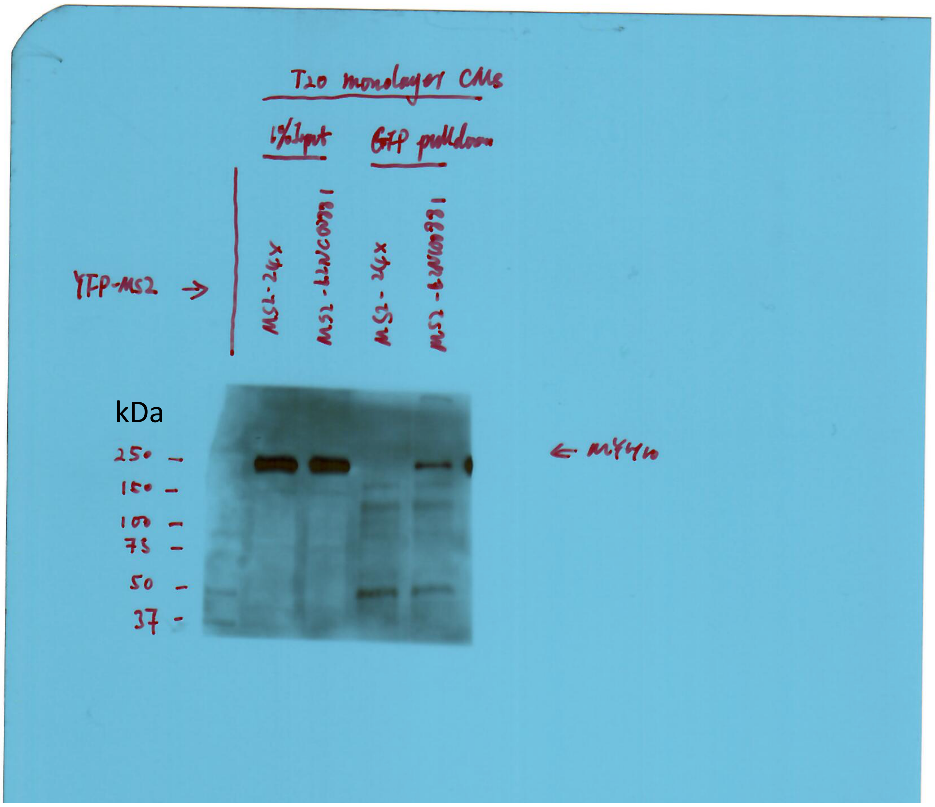

Raw image of Figure 5p left panel

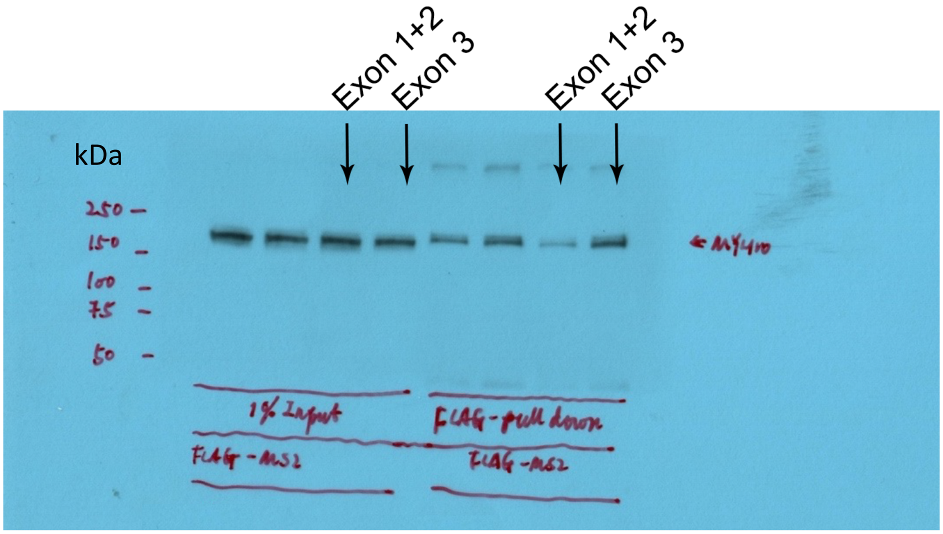

Raw images of Figure 5p right panel (Imaged with Bio-Rad Chemidoc imaging system)

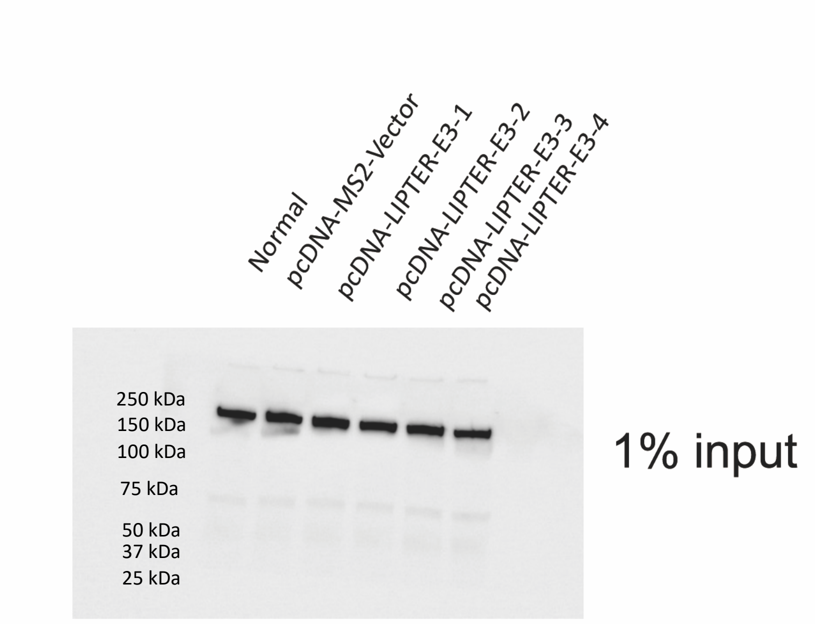

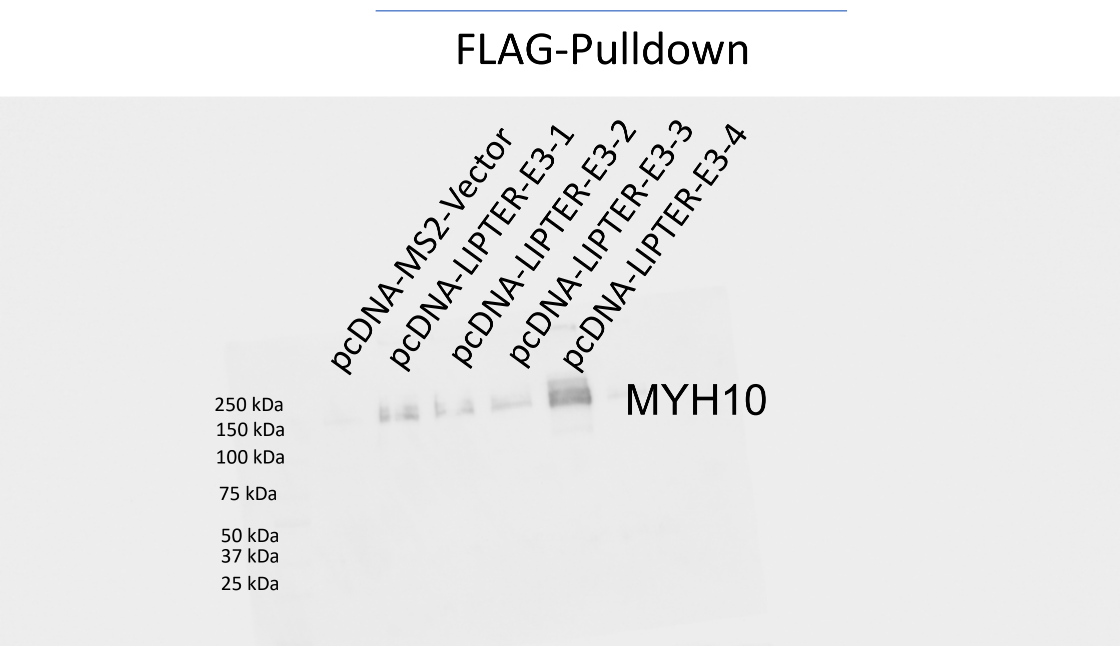

Supplement: Source Data Extended Data Fig. 5 — Unprocessed western blots and/or gels. [file 41556_2023_1162_MOESM23_ESM.pdf]
