# Supplementary figures and images for: Lipid droplet-associated lncRNA LIPTER preserves cardiac lipid metabolism
Source: Nat Cell Biol. 2023 Jun 1;25(7):1033–46. doi: 10.1038/s41556-023-01162-4 (PMC10344779; doi:10.1038/s41556-023-01162-4)

Raw images of Extended Data Figure 2b (Imaged with Bio-Rad Chemidoc imaging system)

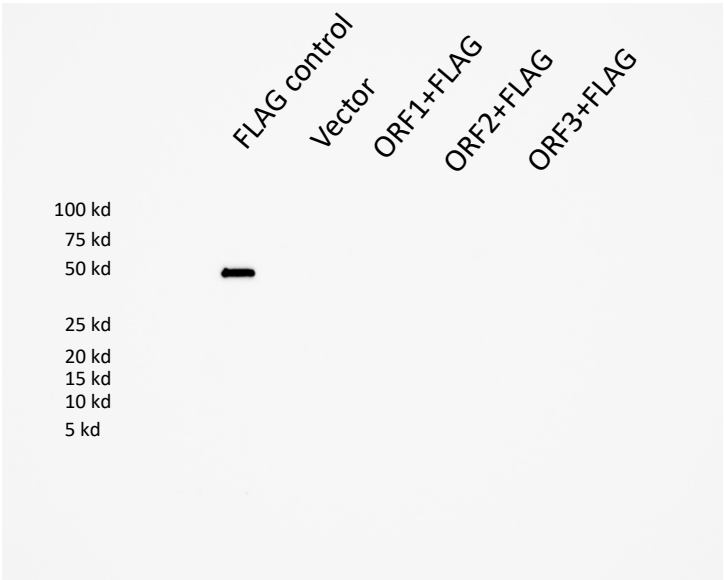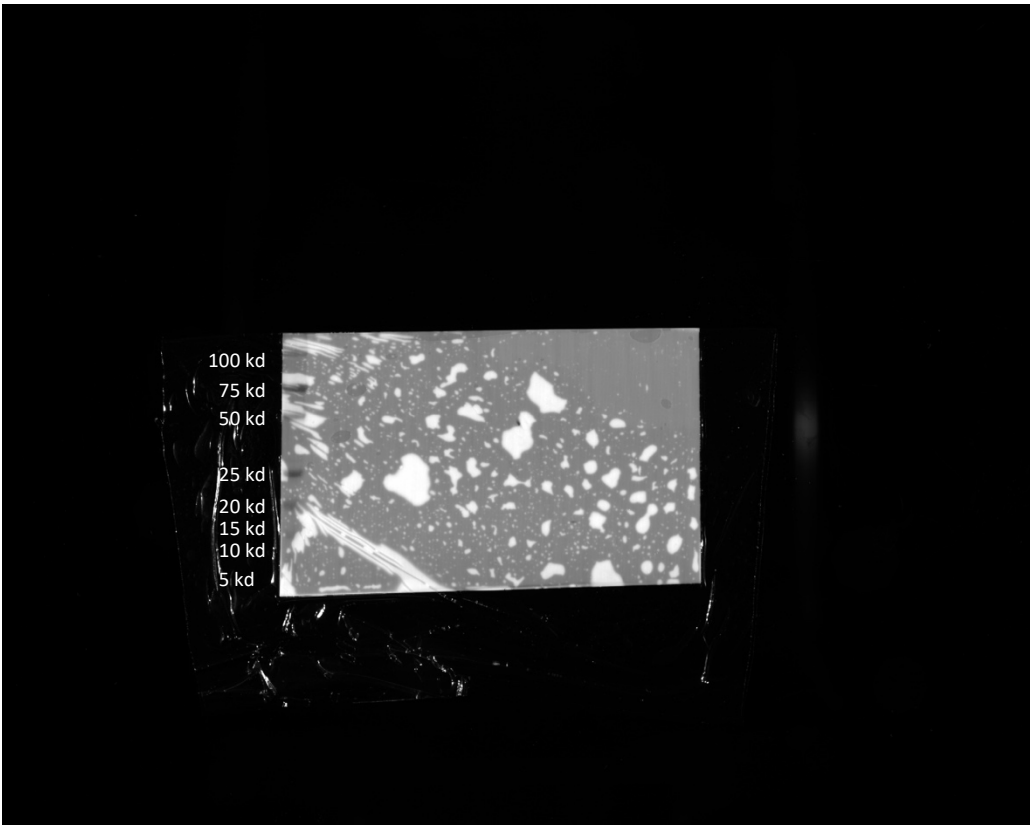

Supplement: Source Data Extended Data Fig. 2 — Unprocessed western blots and/or gels. [file 41556_2023_1162_MOESM24_ESM.pdf]

Raw image of Extended Data Figure 4b (Imaged with Bio-Rad Chemidoc imaging system)

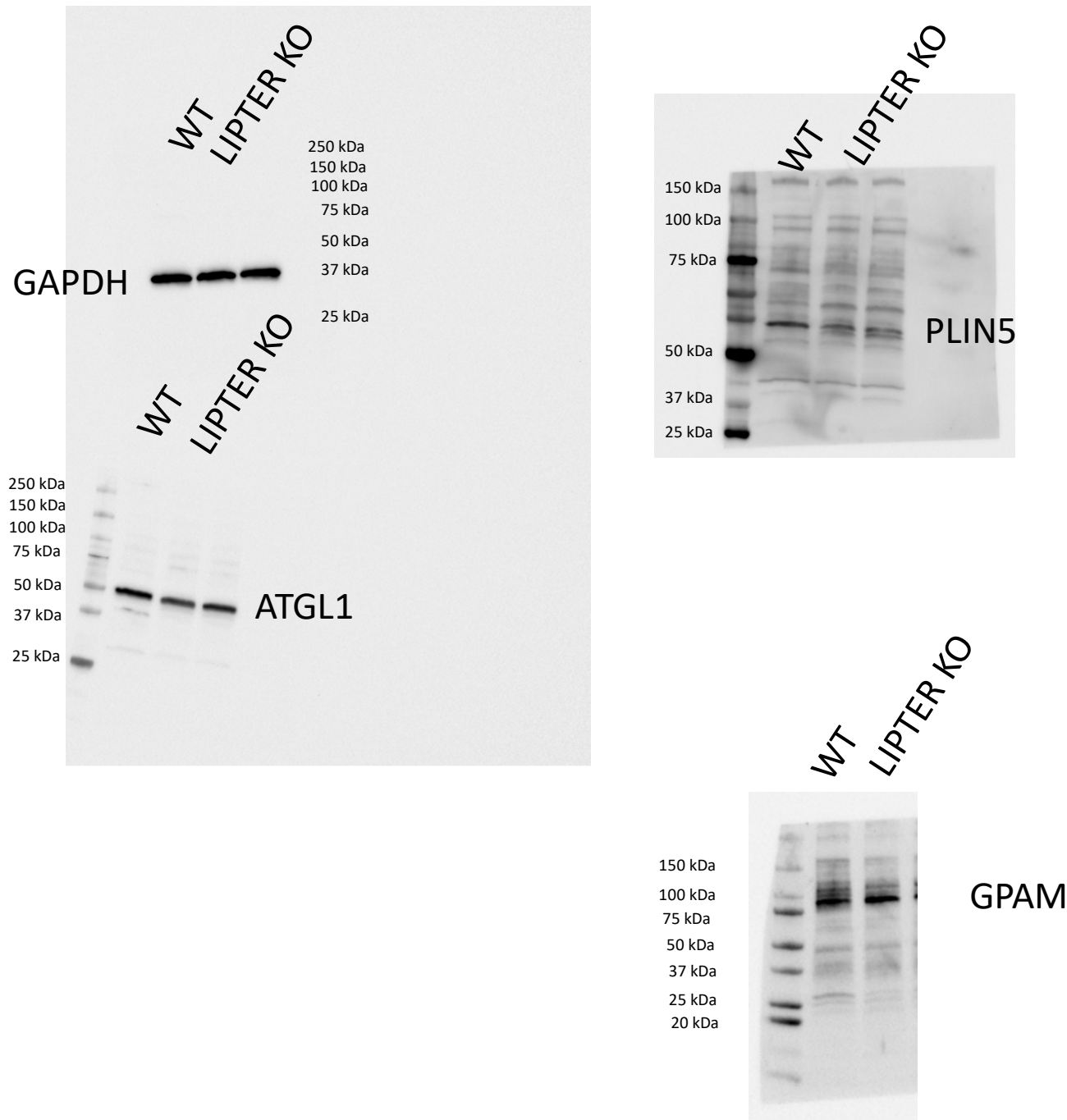

Supplement: Source Data Extended Data Fig. 4 — Unprocessed western blots and/or gels. [file 41556_2023_1162_MOESM25_ESM.pdf]

Raw image of Extended Data Figure 7c silver staining

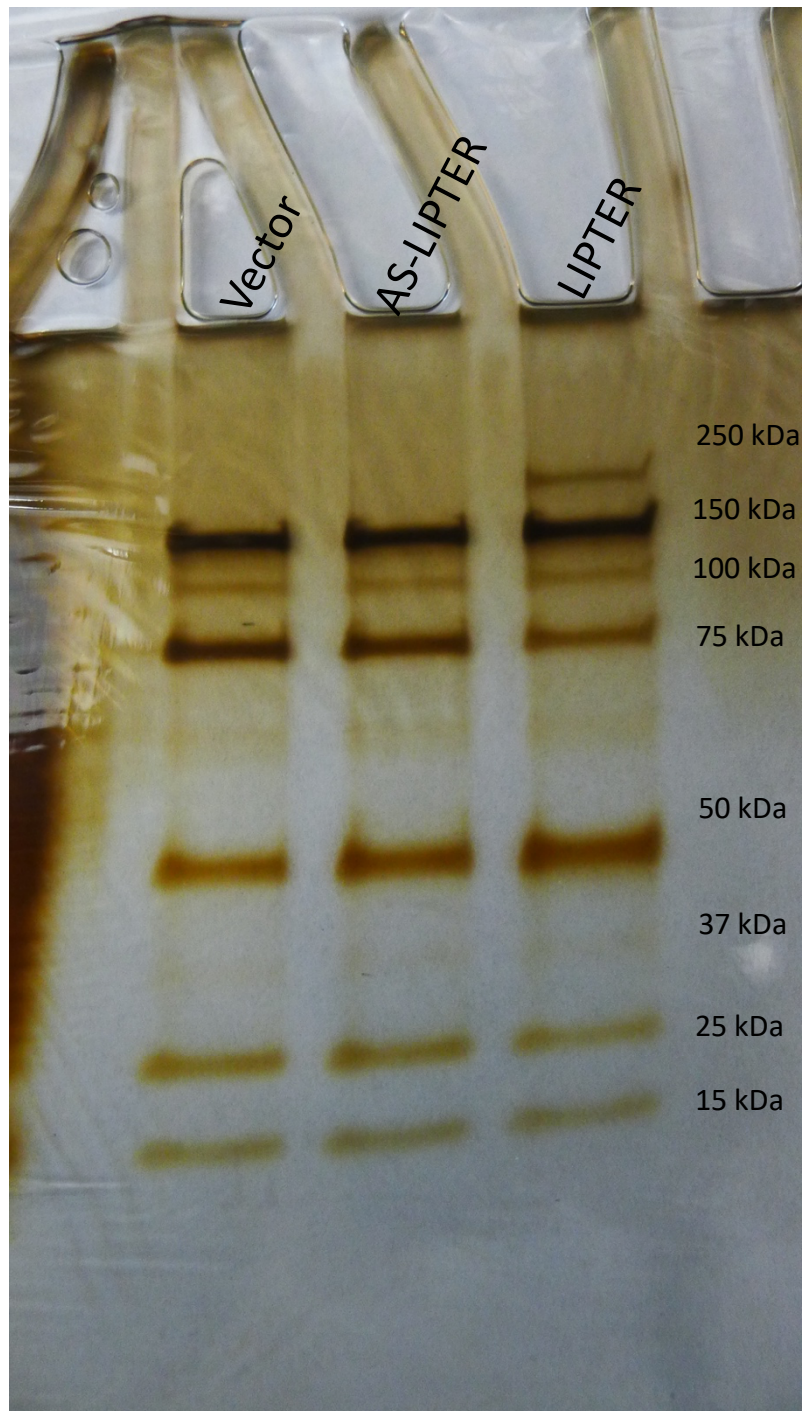

Raw image of Extended Data Figure 7d

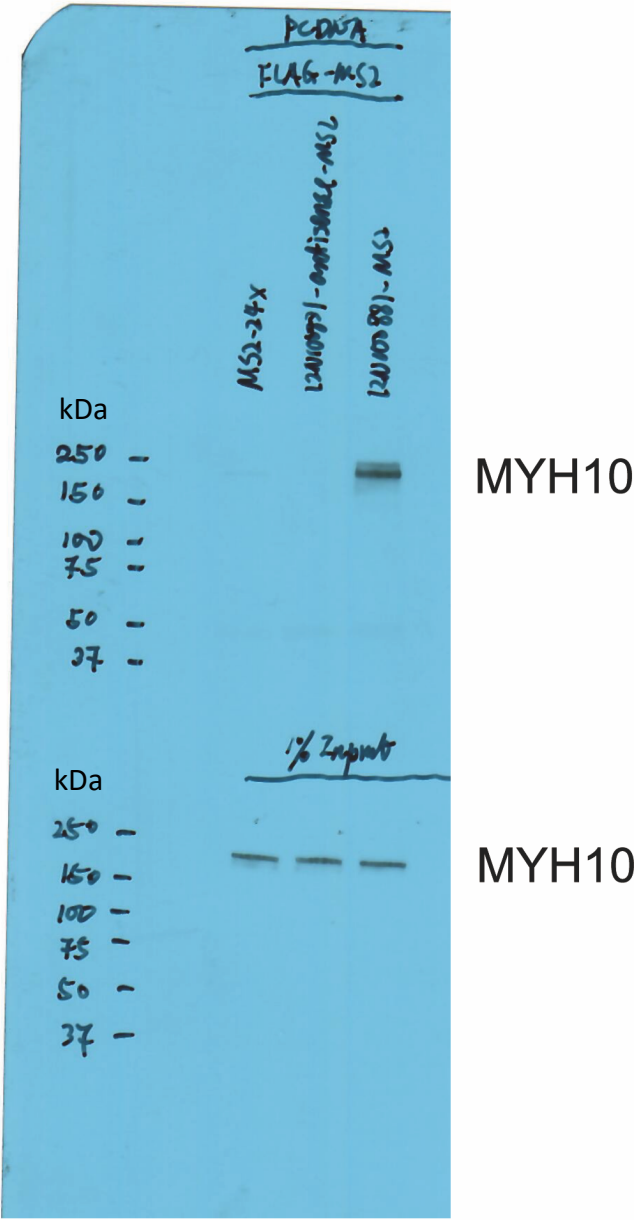

Supplement: Source Data Extended Data Fig. 7 — Unprocessed western blots and/or gels. [file 41556_2023_1162_MOESM26_ESM.pdf]
